# Towards a New Generation of Hormone Therapies: Design, Synthesis and Biological Evaluation of Novel 1,2,3-Triazoles as Estrogen-Positive Breast Cancer Therapeutics and Non-Steroidal Aromatase Inhibitors

**DOI:** 10.3390/ph17010088

**Published:** 2024-01-09

**Authors:** Huda R. M. Rashdan, Mohamad T. Abdelrahman, Anna Chiara De Luca, Maria Mangini

**Affiliations:** 1 Chemistry of Natural and Microbial Products Department, Pharmaceutical and Drug Industries Research Institute, National Research Centre, 33 El Buhouth St, Dokki, Giza 12622, Egypt; 2 Radioisotopes Department, Nuclear Research Centre, Egyptian Atomic Energy Authority, Cairo 13759, Egypt; mohamadt.abdelrahman@gmail.com; 3 Laboratory of Biophotonics and Advanced Microscopy, Institute of Experimental Endocrinology and Oncology “G. Salvatore”, Second Unit, National Research Council, 80131 Naples, Italy; annachiara.deluca@cnr.it

**Keywords:** 1,2,3-triazoles, aromatase inhibitors, breast cancer, hormone therapy, gelatin degradation, RNA extraction and real-time qPCR

## Abstract

Aromatase inhibitors (AIs) show promising features as drugs to treat estrogen-responsive breast cancer as they block aromatase activity, the key enzyme in estrogen synthesis. The current AIs approved by the Food and Drug Administration for breast cancer treatment present severe adverse effects. For these reasons, it is important to develop of new AIs that are more specific and sensitive. In this paper, we report the synthesis and the characterization of new nonsteroidal aromatase AIs containing triazoles moieties for the treatment of hormone-dependent breast cancer in post-menopausal women. A new series of 1,2,3-triazole based molecules were successfully synthetized and their chemical structures were determined from the spectral data (FT-IR, ^13^C NMR, ^1^H NMR, mass spectroscopy) and micro-analytical data. Additionally, the physical properties of the newly synthesized derivatives were reported. The novel compounds were also tested for their anticancer activity in both breast cancer (MCF7 and T-47D) and normal breast (MCF 10A) cell lines, evaluating their effect on cell proliferation, migration, and invasion. The results revealed that the compounds exhibited promising and specific anti-cancer action.

## 1. Introduction

Breast cancer (BC) affects approximately 2.5 million women annually and is the most diagnosed cancer type in women. BC is just second to lung cancer as the leading cause of cancer-related death in women from different age groups around the world. About 70% of patients develop a hormone-responsive BC expressing a high level of estrogen receptors (ERs) [1,2,3]. The risk of developing an estrogen-responsive cancer is higher in postmenopausal women owing to the estrogen production in peripheral tissues [4,5]. In these patients, estrogen plays a key role in promoting cancer cell growth, invasion and metastatization [6,7]. In particular, it can promote cancer onset and progression in two different ways: (1) acting as a “mitogen” [8,9,10,11], stimulating breast tissue to increase mitosis, which can sometimes lead to errors during cell division, resulting in mutations that can cause cancer; (2) some estrogen metabolites can act as carcinogens, damaging DNA and causing cancer [12,13,14]. Moreover, a high level of estrogens in plasma has been associated with a high risk of recurrence and metastasis in ER-positive breast cancer patients.

It was demonstrated that estrogen is biosynthesized via demethylation and aromatization of androgens by aromatase (CYP19), which is the rate-limiting enzyme of these reactions. Aromatase is a member of the P450 family (monooxygenase heme proteins) [15,16]. It is a microsomal enzyme complex formed by two fundamental units: one is the flavoprotein Nicotinamide Adenine Dinucleotide Phosphate-cytochrome P450 reductase (NADPH) and the other is the hemoprotein CYP19.

There are two fundamental strategies to block, manage, and control the progression of the hormone-dependent types of BC: (1) blocking the ER pathway using antagonists or degraders or (2) inhibiting estrogen production using aromatase inhibitors (AIs) [17,18,19,20,21,22]. Tamoxifen (TAM) is a selective ER antagonist largely used in ER-positive BC. TAM can reduce both the BC recurrence rate by 40–50% and the BC-associated mortality [16,23,24,25]. However, BC cells can easily develop TAM resistance, and TAM side effects include an increased risk of endometrial cancer and liver abnormalities [26,27,28]. The AIs were found to have less side effects than the ER antagonists because of the lack of estrogenic effect on the vasculature and uterus, and they currently represent the first line of treatment in post-menopausal women with ER-positive BC [17,29]. AIs can be categorized into two main groups according to their mechanisms of action. Type 1 is the steroidal AIs such as exemestane and formestane, which irreversibly inhibit the aromatase enzyme activity. The second type is the non-steroidal AIs, such as letrozole [21,27,30,31], vorozole and anastrozole, which have already been approved by the Food and Drug Administration (FDA) [10,11,12]. The core structure of non-steroidal AIs is the triazoles [7,8,9] as the nitrogen atom of the triazole moiety plays a fundamental role in the aromatase functionality by interacting with the iron ions of the heme group. In general, non-steroidal AIs possess numerous advantages as they inhibit the last step of estrogen synthesis without any inhibitory effects on the synthesis of other hormones produced via the same pathway. These compounds can block the activity of aromatase in a reversible manner and, comparing the results of trials in which cancer patients were treated with different combination of estrogen receptors modulators (ATAC), the treatment with non-steroidal AIs exhibited a better outcome with significant clinical effects. Despite the fact that non-steroidal AIs are clinically successful, their long-term usage can cause reproductive problems, osteoporosis, androgenic side effects, and joint pain. In addition, these molecules partially inhibit the cytochromes 2D6, 2C8/9, 1A1, 1A2, and 3A4, which are essential in the metabolism of xenobiotics, which increase the drug–drug interaction. Consequently, there is still a need for the development of AIs that demonstrate complete estrogen suppression at doses that have no significant toxicity or no specific side effects.

Herein, we present the synthesis and chemical/physical characterization of new triazole-containing compounds inhibiting aromatase activity. The newly synthetized compounds were tested in vitro for their anti-cancer activity, showing promising features and the potential to be used in vivo as anti-BC drugs.

## 2. Results and Discussion

### 2.1. Chemical Synthesis and Characterization of the Compound ***1***, ***3***, ***4***, ***5***, ***6*** and ***7***

Refining linker combination between the different bioactive heterocycles and 1,2,3-triazoles is considered to boost their efficacy for anticancer application. In order to achieve this, 1-azido-4-methoxybenzene was utilized to synthesize 1-(1-(4-methoxyphenyl)-5-methyl-1*H*-1,2,3-triazol-4-yl)ethan-1-one (**1**) via its reaction with pentane-2,4-dione through condensation reaction in the presence of sodium ethoxide using boiling ethanol as a solvent to afford the desired acetyl triazole **1**. The chemical structure of the acetyl triazole **1** was affirmed by its spectral and micro-analytical data. The FT-IR spectrum of the acetyl triazole **1** exhibited a strong absorption band at 1680 cm^−1^ for the carbonyl group. Its ^1^H NMR spectrum showed two singlet signals at 2.34 and 2.49 ppm, representing the two methyl groups of protons along with a singlet signal at 3.73 ppm for the OCH_3_ protons. Additionally, the aromatic protons provided two doublet signals at *δ* 7.03 and 7.40 ppm with a coupling constant of 10 Hz. The ^13^C-NMR spectrum of the acetyl triazole **1** exhibited significant signals at 9.57 and 27.44 ppm assigned to two methyl groups, a signal at 55.58 ppm for the methoxy group carbon and a signal at 193.27 ppm for the carbonyl carbon. The mass spectrum had a molecular ion peak at *m*/*z* = 231(M^+^, 18%), which supported its molecular formula (C_12_H_13_N_3_O_2_).

The structure of compound **1** was also confirmed by chemical transformation in which it was employed as a key molecule for the synthesis of new heterocyclic compounds containing the 1,2,3-triazole moiety, where it submitted to react with methyl hydrazinecarbodithioate **2** to afford the corresponding derivative methyl (2-(1-(1-(4-methoxyphenyl)-5-methyl-1*H*-1,2,3-triazol-4-yl) ethylidene) hydrazine-1-carbodithioate (**3**). The chemical structure of compound **3** was estimated utilizing its spectral data. The FT-IR spectrum of compound **3** revealed strong absorption bands at 3337 cm^−1^ for the NH group and at 1687 cm^−1^ for the C=O group.

The ^1^H-NMR spectrum of compound **3** exhibited three characteristic signals at 2.45, 2.50 and 2.51 ppm for the three methyl group protons. Additionally, it showed a significant signal at 3.80 ppm for the methoxy group protons and two doublet signals at 7.09 and 7.48 ppm for the aromatic protons with a coupling constant value of *J* = 10.0 Hz and singlet signal at 12.45 ppm for the NH proton. In this regard, its ^13^C-NMR spectrum showed significant signals at 10.70, 14.83 ppm and 17.12 for three methyl groups, a signal at 55.56 ppm for the methoxy group carbon and a signal at 199.63 ppm for the C=S carbon as well as aromatic carbon signals at 114.67, 126.90, 128.24, 133.65, 141.37, 148.19 and 160.05 ppm.

**Scheme 1:**
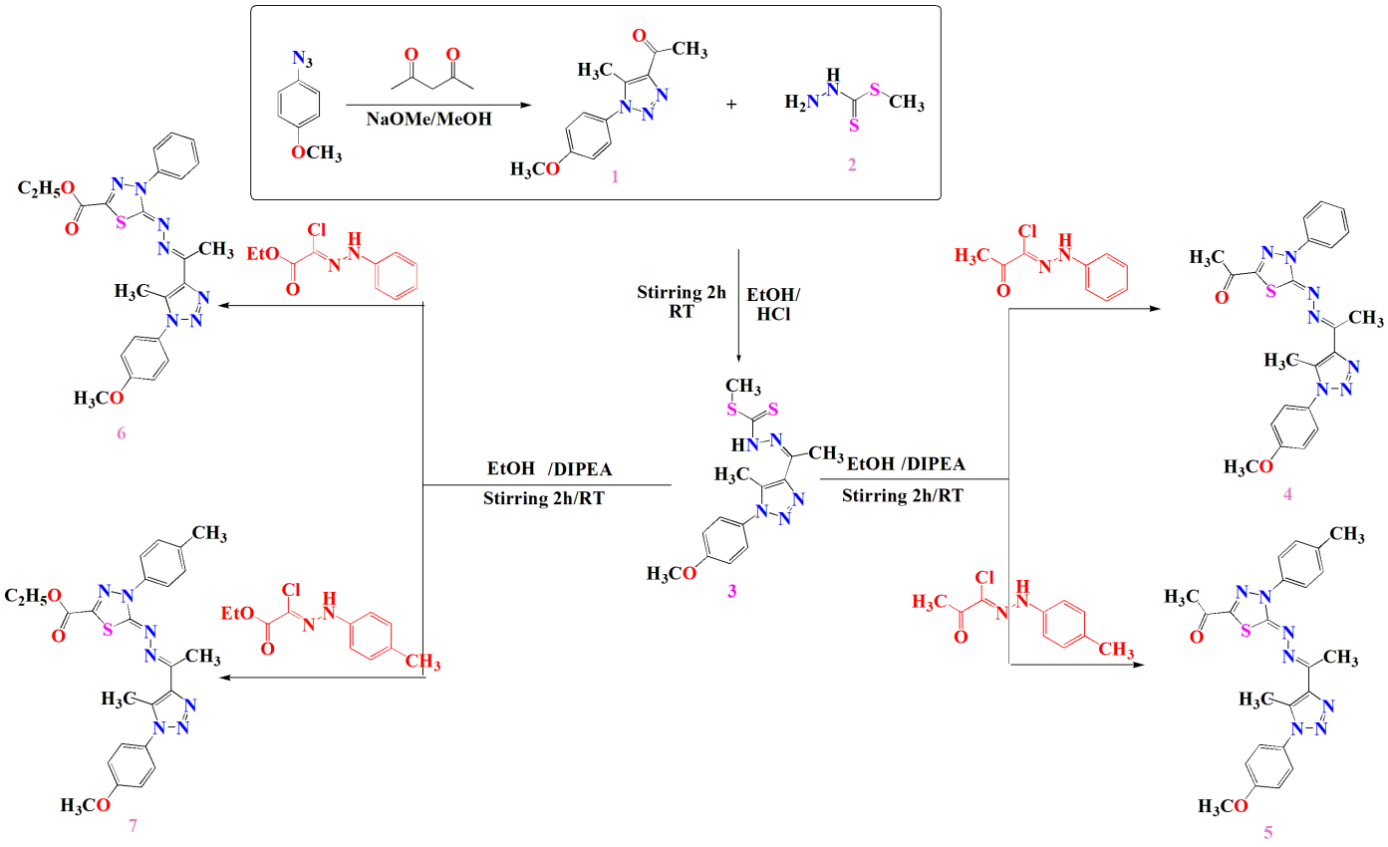
Synthetic procedures of compounds **3**, **4**, **5**, **6** and **7**.

Furthermore, compound **3** was utilized for the preparation of new 1,3,4-thiadiazoles-1,2,3-triazole hybrids via the reaction of compound **3** with the appropriate quantity of hydrazonoyl halides to afford the four 1,3,4-thiadiazole derivatives (**4–7**) as illustrated in Scheme 1.

The chemical structures of thiazdiazole derivatives **4–7** were confirmed by spectral data. For instance, the ^1^H-NMR spectrum of compound **4** revealed characteristic signals at 2.60, 2.63 and 2.73 ppm for three methyl group protons, a singlet signal at 3.89 ppm for the methoxy group protons and a multiplet signal at 7.05 to 8.12 ppm for the aromatic protons. Its ^13^C-NMR spectrum showed significant signals at 11.78, 16.11 and 25.15 ppm for three methyl groups, a signal at 55.72 ppm for the methoxy group carbon and a signal at 189.98 ppm for the C=O carbon in addition to the aromatic carbons’ signals from 114.77 to 164.55 ppm. Analogously, the compound **5** ^1^H-NMR spectrum exhibited characteristic signals at 2.42, 2.59, 2.62 and 2.68 ppm for four methyl group protons, a singlet signal at 3.89 ppm attributed to the methoxy group protons and a multiplet signal extended from 7.05 to 7.96 ppm representing the aromatic protons. Additionally, its ^13^C-NMR spectrum showed significant signals at 11.79, 16.05, 21.14 and 25.13 ppm for four methyl groups, a signal at 55.72 ppm for the methoxy group carbon and a signal at 189.99 ppm for the C=O carbon in addition to the aromatic carbon signals from 114.77 to 164.70 ppm.

Furthermore, the ^1^H-NMR spectrum of compound **6** exhibited a characteristic triplet signal at 1.40 ppm for the methyl protons (COOCH_2_**CH_3_**), two singlet signals at 2.60 and 2.69 ppm representing the two methyl group protons and a singlet signal at 3.89 ppm for the methoxy group protons in addition to a quartet signal at 4.43 ppm for the CH_2_ group protons (COO**CH_2_**CH_3_) and a multiplet signal at 7.05 to 8.10 ppm for the aromatic protons. Its C^13^-NMR spectrum showed significant signals at 11.77 and 14.27 ppm for the methyl groups, a signal at 55.73 attributed to the methoxy group carbon and a signal at 63.02 for the CH_2_ group. It also revealed a characteristic signal at 160.57 ppm for the carbonyl ester in addition to the aromatic carbon signals from 114.76 to 159.01 ppm and a signal at 164.29 ppm for the C=N carbon.

Analogously, the ^1^H-NMR spectrum of compound **7** showed a triplet signal at 1.40 ppm for the methyl protons (COOCH_2_**CH_3_**), two singlet signals at 2.60 and 2.68 ppm representing the two methyl group protons and a singlet signal at 3.89 ppm for the methoxy group protons in addition to a quartet signal at 4.42 ppm for the CH_2_ group protons (COO**CH_2_**CH_3_) and four doublet signals at 7.05, 7.25, 7.38 and 7.93 ppm for the aromatic protons. Its ^13^C-NMR spectrum showed significant signals at 11.76 and 14.27 ppm for the methyl groups, a signal at 55.72 attributed to the methoxy group carbon and a signal at 62.96 for the CH_2_ group. It also revealed a characteristic signal at 160.74 ppm for the carbonyl ester in addition to the aromatic carbon signals from 112.75 to 158.65 ppm and a signal at 164.47 ppm for the C=N carbon (see Supporting Information).

### 2.2. Computational Study

**Figure 1:**
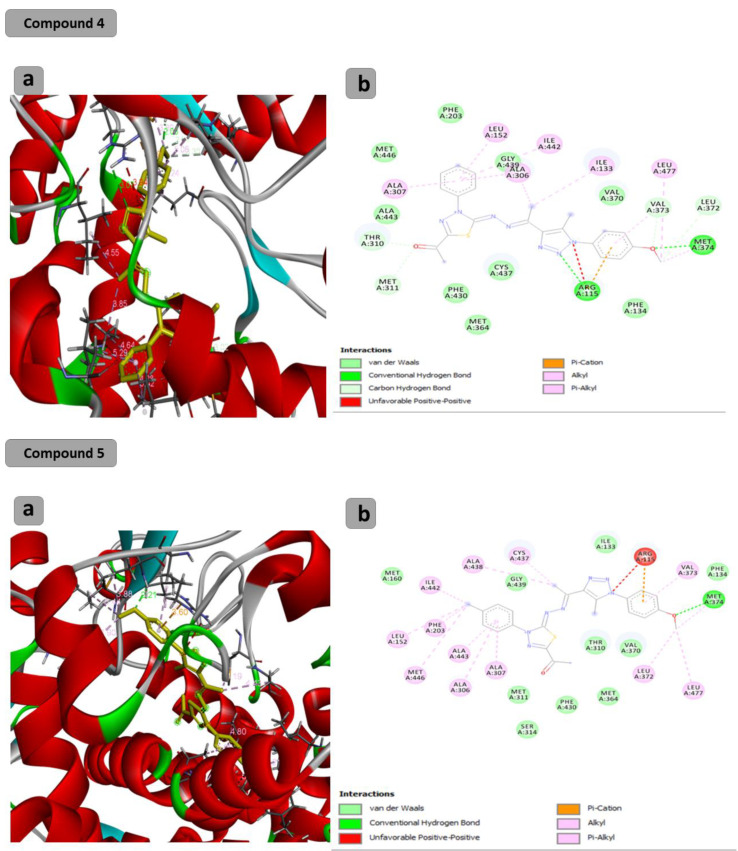

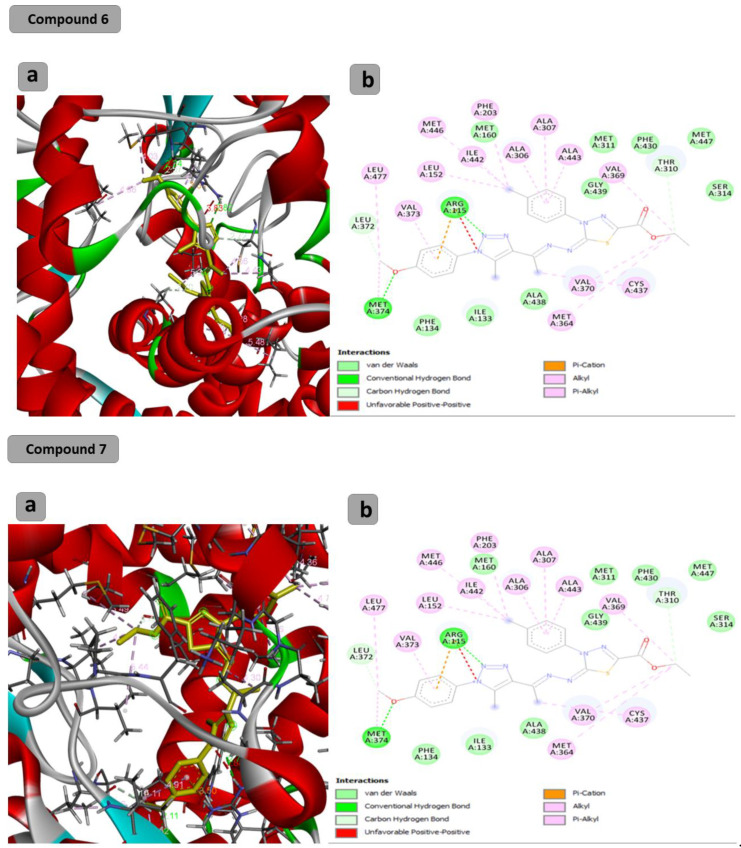
Docking results of synthesized compounds **4**, **5**, **6** and **7** against the target human aromatase enzyme. (**a**) Three-dimensional (3D) and (**b**) two-dimensional (2D) orientations of docked complexes. H-bond interactions are represented by blue and green dotted lines. Pi-stacked interactions are shown as orange lines.

**Figure 2:**
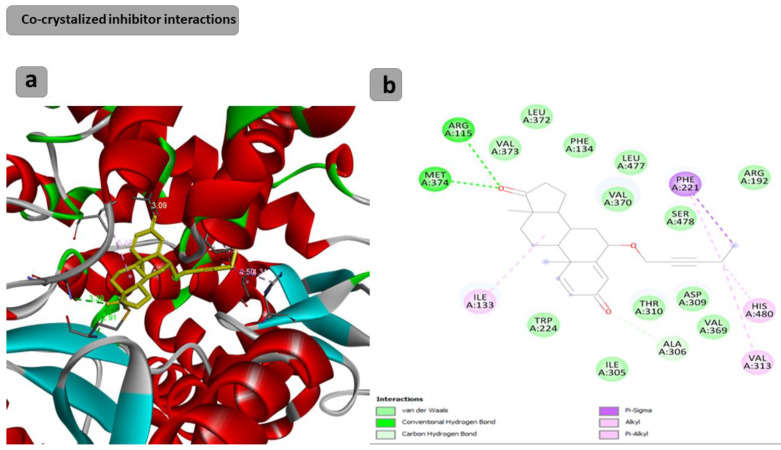
Docking results of the co-crystalized inhibitor interactions with the target human aromatase enzyme. (**a**) Three-dimensional (3D) and (**b**) two-dimensional (2D) orientations of docked complexes. H-bond interactions are represented by blue and green dotted lines. Pi-stacked interactions are shown as orange lines.

The docking results showed that the binding affinity for the docked compounds (**C**) **1**, **3**, **4**, **5**, **6** and **7** was −6.9, −7.0, −9.6, −10.1, −9.7 and −9.8 Kcal/mol, respectively. As shown in Table 1 and Figure 1, both compounds **1** and **3** were able to bind within the Aromatase pocket and form two hydrogen bonds. However, this binding (with amino acids ala438, gly439) is not ideal, and those interactions are different from those formed by the co-crystalized inhibitor (arg115, met374). In the case of the co-crystalized inhibitor (Figure 2), those two H bonds (with Arg115 and met374) help the inhibitor to precisely orient and extend smoothly within the hydrophobic pocket of the aromatase. Consequently, it blocks the entry of the substrate from the tight entrance between the two amino acid residues ser478 and thr310. In the case of compounds **1** and **3,** they had different orientations with two different H bonds. As shown in Figure 1 and Figure 2, compounds **4**, **5**, **6** and **7** could form two H bonds similar to those formed by the co-crystalized inhibitor (arg115, met374) and had the chance to block the entry of the substrate. For compound **7** (Figure 1), a high number of interactions were revealed that could stabilize the compound within the aromatase active site and interacted with one of the gate keeper’s amino acids (thr310) by forming C–H interaction in a manner that blocks the entry of the aromatase substrate from the gate between (ser478 and thr310) and consequently inhibit the targeted aromatase enzyme. In the case of the co-crystalized inhibitor (Figure 2), those two H bonds help the inhibitor to block the entry of the substrate between the two AA residues ser478 and thr310, while in the case of **C1** and **C3,** they had different orientations with two different H bonds. As shown in Figure 1 and Figure 2, **C4**, **C5**, **C6** and **C7** could form two H bonds similar to those formed by the co-crystalized inhibitor (arg115, met374) and have the chance to block the entry of the substrate. **C7** (Figure 1) was observed to have a high number of interactions that can stabilize the compound within the aromatase active site and interacted with one of the gate keeper’s amino acids (thr310) by forming C–H interaction in a manner that blocks the entry of the aromatase substrate from the gate between ser478 and thr310 and consequently inhibits the targeted aromatase enzyme.

The docking studies revealed that **C4**, **C5**, **C6** and **C7** have higher binding affinity and interact with vital AA residues within the aromatase pocket, so they can be recommended for the in vitro testing of their anti-cancer activity.

### 2.3. In Vitro Analysis of the Aromatase Activity Inhibition by ***C4***, ***C5***, ***C6*** and ***C7***

**Figure 3:**
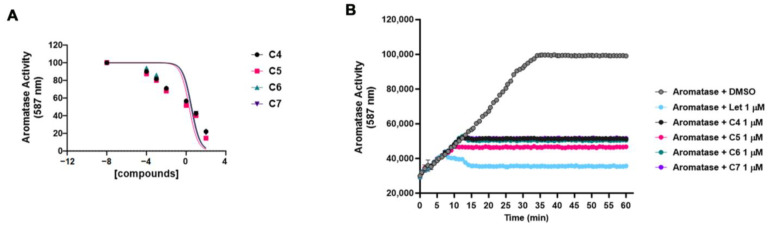
Compound **4**, **5**, **6** and **7** inhibitions of the aromatase activity in vitro. (**A**) The aromatase enzymatic activity was measured without and with increasing concentration (from 0 to 100 μM) of compounds **4** (**C4**), **5** (**C5**), **6** (**C6**) and **7** (**C7**) using a fluorogenic enzymatic assay (see Section 3). The graph shows the dose–response of log10 concentrations of **C4** (black dot), **C5** (pink square), **C6** (green triangle) and **C7** (purple triangle) versus normalized fluorescence intensity (587 nm). The IC_50_ values were calculated and reported in the Section 2. Data are shown as mean ± SDs of three independent experiments. (**B**) The aromatase activity was measured alone (with DMSO) or in combination with 1 μM letrozole (Let), compounds **4** (**C4**), **5** (**C5**), **6** (**C6**) and **7** (**C7**) throughout the time up to 60′. Data are shown as mean ± SDs of three independent experiments.

Based on the results of the docking experiments, we tested compounds **C4**, **C5**, **C6** and **C7** in vitro for their ability to inhibit the aromatase enzymatic activity, calculating the half-maximal inhibitory concentration (IC_50_). The aromatase activity was measured using a fluorogenic enzymatic assay (see Section 3) in the presence of an increasing (from 0 to 100 μM) concentration of the four compounds (Figure 3A). **C4** showed an IC_50_ of 3.62 μM, **C5** showed an IC_50_ of 1.991 μM, **C6** showed an IC_50_ of 3.57 μM, and **C7** showed an IC_50_ of 2.993 μM. These data were in line with the docking analysis results, with C5 being the most powerful compound both in the docking experiments and in inhibiting the aromatase activity in vitro. Figure 3B shows the inhibition of the aromatase activity by the four compounds at 1 μM throughout the time up until 60′. The activity of the four compounds was compared to that of letrozole, a well-known and largely used non-steroidal aromatase inhibitor. The four compounds were able to inhibit aromatase activity by about 50%.

### 2.4. Anticancer Assays

The anticancer activities of compounds **C4**, **C5**, **C6** and **C7** have been tested by analyzing the BC cell proliferation, migration, and invasiveness.

**Figure 4:**
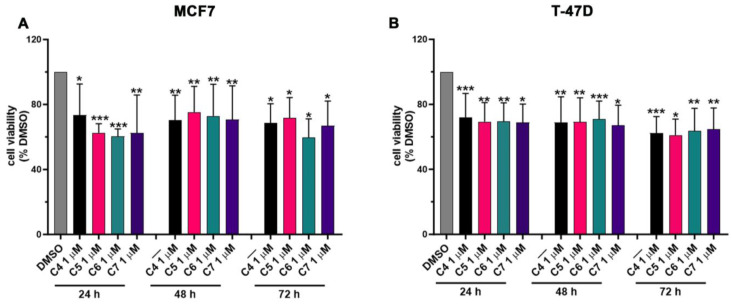
Effects of compounds **4**, **5**, **6** and **7** on MCF7 and T-47D cell proliferation. Cell viability was assessed using an MTT assay in MCF7 (**A**) and T-47D (**B**) cells not treated (DMSO) or treated with 1 μM of compounds **4** (**C4**), **5** (**C5**), **6** (**C6**), and **7** (**C7**) for 24, 48, and 72 h. Data are shown as mean ± SDs of six independent experiments. * *p* < 0.05; ** *p* < 0.01; *** *p* < 0.005 vs. DMSO (Student’s *t*-tests).

ER-positive BC growth and progression strictly rely on the estrogen levels and on the aromatase activity in synthetizing estrogens de novo. In order to investigate the effect of **C4**, **C5**, **C6** and **C7** on BC cell proliferation, MCF7 and T-47D cells were used since these cell lines express high levels of aromatase, showing an aromatase-dependent proliferation. Cells were treated with an increasing concentration of the four compounds (from 0 to 100 μM) for 24, 48 and 72 h (h), and the cell viability was assessed by performing an MTT assay (Figure S1). The addition of the compounds resulted in a dose- and time-dependent decrease in cancer cell viability (Figure S1). The IC_50_ values were calculated for each cell line, compound and time point and are shown in Table 2 and Table 3. Based on these results, we chose to treat cancer cells with 1 μM of **C4**, **C5**, **C6** and **C7** for 24 h in the successive experiments, aiming to further investigate the anti-cancer effects of the four compounds. Figure 4 shows the viability of MCF7 (Figure 4A) and T47D (Figure 4B) under treatment with 1 μM of **C4**, **C5**, **C6** and **C7** for 24 h. In particular, the four compounds were able to reduce the cancer cell proliferation by about 25–30%. As a control, normal breast cells (MCF 10A) were also treated with the compounds at the same concentration for the same time points, and an MTT assay was carried out. Crucially, **C4**, **C5**, **C6** and **C7** showed no significant effects on MCF 10A proliferation (Figure S2).

These results suggest that **C4**, **C5**, **C6** and **C7** can specifically inhibit aromatase-dependent BC cell proliferation and show no or not significant effects on normal breast cells.

**Figure 5:**
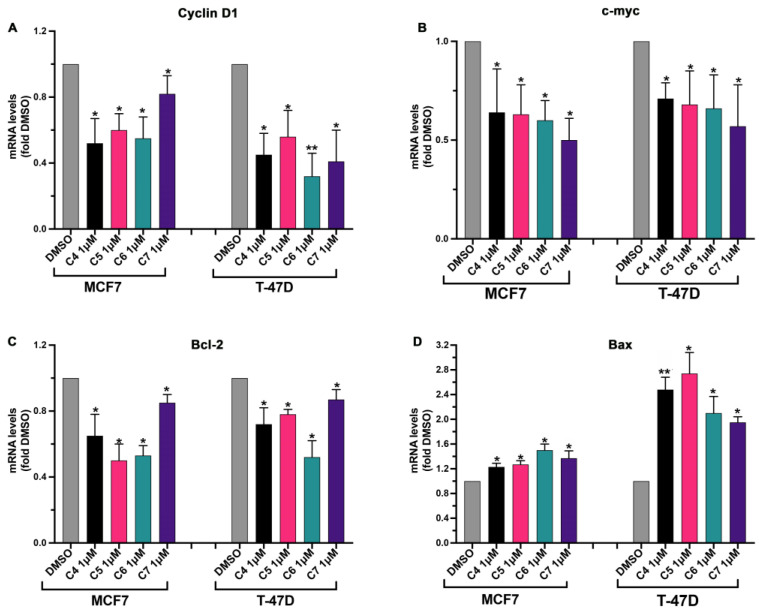
Effects of compounds **4**, **5**, **6** and **7** on cell-proliferation-related genes in MCF7 and T-47D cells. MCF7 and T-47D cells were plated as described in the Section 3 and treated with DMSO or with compounds **4** (**C4**), **5** (**C5**), **6** (**C6**) and **7** (**C7**) at 1 μM for 24 h. After the treatment, Cyclin D1 (**A**), c-myc (**B**), Bcl-2 (**C**) and Bax (**D**) gene expression were quantified using real-time qPCR and normalized using GAPDH expression as the housekeeping gene. Data are shown as mean ± SDs of three independent experiments. * *p* < 0.05, ** *p* < 0.01 vs. DMSO (Student’s *t*-tests).

In order to deepen our understanding of the mechanism through which the four AIs affect cancer cell proliferation, the expression of genes involved in cell cycle progression and apoptosis was checked in cells treated or not with the four compounds. Cyclin D1 mRNA expression was tested since this protein is a mitogen-regulated cell-cycle control element that is over-expressed in ER-positive BC playing a key role in cancer growth and progression [32], and a higher expression of Cyclin D1 has been linked to a higher mortality rate in ER-positive BC patients [32]. C-myc gene expression was, additionally, checked under the effect of **C4**, **C5**, **C6** and **C7** since it has been largely reported that this transcriptional factor regulates the expression of several genes involved in BC onset and progression and that its over-expression has been linked to aromatase inhibitor resistance development in BC [22]. Moreover, the expressions of Bax and Bcl-2 were checked given that they are pro- and anti-apoptotic proteins, respectively, and their involvement in BC progression has been reported [33]. Additionally, in this case, both BC (MCF7 and T-47D) and normal breast (MCF 10A) cell lines were used. Cells were treated with 1 μM of the four compounds for 24 h, and then gene transcription was assessed using real-time qPCR. The four compounds did not affect the expression of the analyzed genes in normal breast cells (Figure S3). On the contrary, the four compounds were able to modulate the expression of the selected genes in the BC cell lines. As expected, the expression of the gene involved in the promotion of cell proliferation (Cyclin D1, c-myc, Bcl-2) was inhibited by the four compounds (Figure 5A–C) while gene expression of the pro-apoptotic Bax was enhanced by the treatment with the four compounds (Figure 5D).

Altogether, these results indicate that our compounds are active in inhibiting aromatase-dependent BC cell growth, affecting the expression of proliferation-related genes.

### 2.5. Compounds ***4***, ***5***, ***6*** and ***7*** Inhibit Cancer Cell Migration and Invasiveness

The main cause of death in BC patients is metastasis in distant organs [34]. The first step of the metastatization process is epithelial–mesenchymal transition, a biological process through which cancer cells gain enhanced migratory capacity and the ability to degrade the extracellular matrix [35]. The estrogen-activated signaling pathway positively contributes to the metastatic process in BC through the activation of stimulatory processes that promote the onset, establishment and progression of metastasis [36]. We investigated the ability of **C4**, **C5**, **C6** and **C7** in reducing the aromatase-dependent BC migratory and degradation capacity.

**Figure 6:**
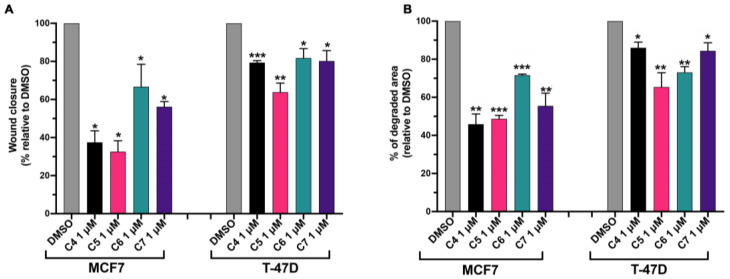
Compounds **4**, **5**, **6** and **7** inhibit breast cancer cell migratory and invasion activity. (**A**) MCF7 and T-47D cells were plated as described in the Section 3. After making the wound, cells were treated with DMSO or with compounds **4** (**C4**), **5** (**C5**), **6** (**C6**) and **7** (**C7**) at 1 μM for 24 h in media supplemented with 0.5% FBS. After the treatment, the wound closure was calculated with respect to the untreated cells (DMSO). Data are shown as mean ± SDs of three independent experiments. * *p* < 0.05; ** *p* < 0.01; *** *p* < 0.005 vs. DMSO (paired Student’s *t*-tests). (**B**) MCF7 and T-47D cells were plated as described in the Section 3 and treated with DMSO or with compounds **4** (**C4**), **5** (**C5**), **6** (**C6**) and **7** (**C7**) at 1 μM for 24 h. After the treatment, the degraded area was calculated with respect to the untreated cells (DMSO). Data are shown as mean ± SDs of three independent experiments. * *p* < 0.05; ** *p* < 0.01; *** *p* < 0.005 vs. DMSO (paired Student’s *t*-tests).

To investigate the BC migratory capacity, MCF 10, MCF7 and T-47D cells were seeded in a 96-well plate, and once they reached ~100% of confluence, a scratch was made as described in Section 3. Cells were treated with 1 μM **C4**, **C5**, **C6** and **C7** for 24 h. During the time of the treatment with the compounds, cells were grown in media supplemented with 0.5% FBS in order to slow down cell proliferation, which could contribute to wound closure and affect the final result. The cell migration was measured as the percentage of wound closure with respect to untreated cells (DMSO). **C4**, **C5**, **C6** and **C7** did not affect the migration of healthy cells (Figure S4A). On the contrary, they were able to inhibit BC cell migration. In particular, MCF-7’s ability to close the wound was inhibited by about 70% by **C4** and **C5**, by about 30% by **C6,** and by about 40% by **C6** (Figure 6A). T-47D’s migratory capacity was affected by about 20% by **C4**, **C5** and **C7** and by about 40% by **C5** (Figure 6A).

Fluorescent gelatin degradation assays were carried out to investigate the ability of **C4**, **C5**, **C6** and **C7** to affect the ability of BC cells to degrade the extracellular matrix. Indeed, gelatin can mimic the extracellular matrix that cancer cells must degrade in order to migrate and invade secondary organs. MCF 10A, MCF7 and T-47D cells were plated on fluorescent-gelatin-coated coverslips as described in the Section 3. After 24 h, **C4**, **C5**, **C6** and **C7** were added to cells at 1 μM for 24 h. The percentage of degraded area was calculated with respect to the untreated cells (DMSO). **C4**, **C5**, **C6** and **C7** did not have any effects on MCF 10A degradation activity, which was already lower compared to the BC cell degradation activity (Figure S4B). On the contrary, **C4**, **C5** and **C7** were able to inhibit the MCF7 degradation activity by about 50% while **C6** reduced the gelatin degradation activity of MCF7 by about 30% (Figure 6B). Additionally, T-47D cell extracellular matrix degradation was affected by the treatment with the four compounds; in particular, **C4** and **C7** reduced the degradation activity by about 20%, **C5** by about 35% and **C6** by about 25% (Figure 6B).

Altogether, these results indicate that all the tested compounds were able to inhibit the migratory and degrading BC cell activity, which are necessary and crucial features in the metastatization process.

## 3. Materials and Methods

### 3.1. Chemistry Instrumentation

All the melting points of the as-prepared compounds are uncorrected, determined utilizing an electrothermal device. The FT-IR data were detected (KBr discs) using a Shimadzu FT-IR 8201 PC spectrophotometer. ^1^H- and ^13^C-NMR spectra were determined in DMSO-d_6_ solutions using a BRUKER 500 FT-NMR system spectrometer, and chemical shifts are represented in ppm units utilizing TMS as the internal reference. Additionally, the elemental analyses were performed at the Microanalytical Center of Cairo University. Finally, the mass spectra were detected using a GC-MS QP1000 EX Shimadzu.

### 3.2. Synthesis

#### 3.2.1. 1-(1-(4-Methoxyphenyl)-5-methyl-1H-1,2,3-triazol-4-yl)ethan-1-one (**1**)

Beige crystals. Yield: 56%, m.p.: 217–219 °C, FT-IR (KBr, cm^−1^): *v* 1680(C=O), 1612 (C=N), 1590 (C=C), ^1^H-NMR (DMSO-d*_6_*): *δ* 2.34 (s, 3H, CH_3_), 2.49 (s, 3H, CH_3_), 3.73 (s, 3H, OCH_3_), 7.03 (d, 2H, 2CH), 7.40 (d, 2H, 2CH) ppm;^13^C-NMR (100 MHz, DMSO-d*_6_*): *δ* 9.57, 27.44 (2 CH_3_), 55.58 (OCH_3_), 114.75, 126.66, 127.74, 137.66, 142.65, 160.23 (Ar-C), 193.27 (C=O) ppm; MS *m*/*z* (%): 231 (M^+^, 62). Anal. Calcd. for “C_12_H_13_N_3_O_2_” (231.26): C, 62.33; H, 5.67; N, 18.17; Found: C, 62.37; H, 5.62; N, 18.14%.

#### 3.2.2. Methyl 2-(1-(1-(4-Methoxyphenyl)-5-methyl-1H-1,2,3-triazol-4-yl)ethylidene)hydrazine-1-carbodithioate (**3**)

A mixture of 1-(1-(4-Methoxyphenyl)-5-methyl-1*H*-1,2,3-triazol-4-yl)ethan-1-one (**1**) (1.15 gm, 5 mmol) and methyl hydrazinecarbodithioate **2** (0.6 gm, 5 mmol) in 20 mL ethanol with the addition of 1mL of hydrochloric acid as a catalyst was stirred at room temperature for 2 h, and the reaction was monitored using TLC. The solid was collected and washed with water/ethanol and then recrystallized from the ethanol to give the desired derivative as yellow crystals. Yield: 82%, m.p.: 175–177 °C, FT-IR (KBr, cm^−1^): *v* 3382 (NH), 1620 (C=N), 1590; ^1^H-NMR (DMSO-d*_6_*): *δ* 2.46 (s, 3H, CH_3_), 2.50 (s, 3H, CH_3_), 2.51 (s, 3H, CH_3_), 3.80(s, 3H, OCH_3_), 7.09 (d, 2H, 2CH), 7.48 (d, 2H, 2CH), 12.45 (s, 1H, NH) ppm;^13^C-NMR (100 MHz, DMSO-d*_6_*): *δ* 10.70, 14.83 and 17.12 (3CH_3_), 55.56 (OCH_3_), 114.67, 126.90, 128.24, 133.65, 141.37, 148.19 and 160.05 (Ar-C), 199.63 (C=S) ppm; MS *m*/*z* (%): 335 (M^+^, 17). Anal. Calcd. for “C_14_H_17_N_5_OS_2_” (335.44): C, 50.13; H, 5.11; N, 20.88; Found: C, 50.18; H, 5.06; N, 20.82%.

#### 3.2.3. General Procedures for Synthesis of Compounds **4**–**7**

A mixture of **C3** (1.67 gm, 5 mmol) and the selected derivative of the hydrazonoyl halides (5 mmol) and 2–3 drops of diisopropylethylamine (DIPEA) as a catalyst in 10 mL ethanol was stirred at room temperature for 2 h. Stirring was continued for approximately 5 h, and the reaction was monitored using TLC. The solid was collected and washed with water/ethanol and then recrystallized from the proper solvent to give the desired derivatives **4**–**7**.

#### 3.2.4. 1-(5-((1-(1-(4-Methoxyphenyl)-5-methyl-1H-1,2,3-triazol-4-yl)ethylidene)hydrazono)-4-phenyl-4,5-dihydro-1,3,4-thiadiazol-2-yl)ethan-1-one (**4**)

Yellow crystals. Yield: 76%, m.p.: 264–266 °C, FT-IR (KBr, cm^−1^): *v* 1685 (C=O), 1620 (C=N), 1585; ^1^H-NMR (DMSO-d*_6_*): *δ* 2.60 (s, 3H, CH_3_), 2.63 (s, 3H, CH_3_), 2.73 (s, 3H, CH_3_), 3.89 (s, 3H, OCH_3_), 7.05–8.12 (m, 9H, ArH) ppm;^13^C-NMR (100 MHz, DMSO-d*_6_*): *δ* 11.78, 16.11, 25.15 (3 CH_3_), 55.72 (s, 3H, OCH_3_), 114.77, 115.49, 121.19, 126.89, 128.99, 129.57, 133.78, 139.44, 142.75, 150.46, 158.05, 160.59, 164.55 (Ar-C), 189.98(C=O) ppm; MS *m*/*z* (%): 447 (M^+^, 25). Anal. Calcd. for “C_22_H_21_N_7_O_2_S” (447.52): C, 59.05; H, 4.73; N, 21.91; Found: C, 59.09; H, 4.68; N, 21.85%.

#### 3.2.5. 1-(5-((1-(1-(4-Methoxyphenyl)-5-methyl-1H-1,2,3-triazol-4-yl)ethylidene)hydrazono)-4-(p-tolyl)-4,5-dihydro-1,3,4-thiadiazol-2-yl)ethan-1-one (**5**)

Yellow crystals. Yield: 89%, m.p.: 239–241 °C, FT-IR (KBr, cm^−1^): *v* 1680 (C=O), 1622 (C=N), 1600 (C=C); ^1^H-NMR (DMSO-d*_6_*): *δ* 2.42 (s, 3H, CH_3_), 2.59 (s, 3H, CH_3_), 2.62 (s, 3H, CH_3_), 2.68 (s, 3H, CH_3_), 3.89(s, 3H, OCH_3_), 7.05–7.96 (m, 8H, Ar-H) ppm;^13^C-NMR (100 MHz, DMSO-d*_6_*): *δ* 11.79, 16.05, 21.14, 25.13 (4 CH_3_), 55.72 (OCH_3_), 114.77, 115.44, 122.05, 126.86, 129.60, 130.04, 133.73, 137.07, 142.82, 150.36, 157.76, 160.59, 164.70 (Ar-C), 189.99 (C=O) ppm; MS *m*/*z* (%): 461 (M^+^, 58). Anal. Calcd. for “C_23_H_23_N_7_O_2_S” (461.54): C, 59.85; H, 5.02; N, 21.24; Found: C, 59.82; H, 4.98; N, 21.19%.

#### 3.2.6. Ethyl 5-((1-(1-(4-Methoxyphenyl)-5-methyl-1H-1,2,3-triazol-4-yl)ethylidene)hydrazono)-4-phenyl-4,5-dihydro-1,3,4-thiadiazole-2-carboxylate (**6**)

Yellowish white crystals. Yield: 86%, m.p.: 215–217 °C, FT-IR (KBr, cm^−1^): *v* 1725 (C=O, ester), 1620 (C=N), 1600 (C=C); ^1^H-NMR (DMSO-d_6_): *δ* 1.40 (s, 3H, COOCH_2_CH_3_), 2.60 (s, 3H, CH_3_), 2.69 (s, 3H, CH_3_), 3.89(s, 3H, OCH_3_), 4.43(q, 2H, COOCH_2_CH_3_), 7.05–8.10 (m, 9H, ArH) ppm;^13^C-NMR (100 MHz, DMSO-d_6_): *δ* at 11.77, 14.27 (2CH_3_), 55.73 (OCH_3_), 63.02 (CH_2_), 114.76, 122.13, 126.91, 126.99, 128.95, 133.62, 139.41, 142.63, 142.38, 157.79, 159.01, 160.57, 164.29 (Ar-C), 160.57(C=O), 164.29 (C=N) ppm; MS *m*/*z* (%): 477 (M^+^, 12). Anal. Calcd. for “C_23_H_23_N_7_O_3_S” (477.54): C, 57.85; H, 4.85; N, 20.53; Found: C, 57.89; H, 4.82; N, 20.51%.

#### 3.2.7. Ethyl 5-((1-(1-(4-Methoxyphenyl)-5-methyl-1H-1,2,3-triazol-4-yl) ethylidene) hydrazono)-4-(p-tolyl)-4,5-dihydro-1,3,4-thiadiazole-2-carboxylate (**7**)

Orange crystals. Yield: 92%, m.p.: 210–212 °C, FT-IR (KBr, cm^−1^): *v* 1715 (C=O, ester), 1610 (C=N), 1590 (C=C);^1^H-NMR (CDCL_3_-d*_6_*): *δ* 1.40 (s, 3H, COOCH_2_CH_3_), 2.40 (s, 3H, CH_3_), 2.60 (s, 3H, CH_3_), 2.68 (s, 3H, CH_3_), 3.89 (s, 3H, OCH_3_), 4.42 (s, 2H, COOCH_2_CH_3_), 7.05 (d, 2H, *J* = 5 Hz), 7.25 (d, 2H, *J* = 10 Hz), 7.38 (d, 2H, *J* = 10 Hz), 7.93 (d, 2H, *J* = 5 Hz) ppm;^13^C-NMR (100 MHz, DMSO-d_6_): *δ* at 11.76, 14.27, 16.07 (3CH_3_) 55.72 (OCH_3_), 62.96 (CH_2_), 112.75, 114.82, 122.19, 126.65, 128.71, 129.11, 133.63, 136.99, 138.57, 142.31, 157.08, 158.65 (Ar-H), 160.74 (C=O), 164.47 (C=N) ppm; MS *m*/*z* (%): 491 (M^+^, 45). Anal. Calcd. for “C_24_H_25_N_7_O_3_S” (491.17): C, 58.64; H, 5.13; N, 19.95; Found: C, 58.68; H, 5.09; N, 19.91%.

### 3.3. Computational Study

The human aromatase’ X-ray protein structure was downloaded from the protein data bank (www.rcsb.org) [37] PDB ID: 4gL7. For the protein, water molecules and co-crystalized ligands were removed, then the prepared structure was saved as a PDB file in BioviaDiscoverstudio2021 for further docking steps. The CB-DOCK webserver was used for cavity detection, and molecular docking was accessed via http://clab.labshare.cn/cb-dock/php/ to perform docking with the default standard protocol. The 7 compounds were docked against 4gl7 at a cavity volume of 379, grid box dimensions x: 85.07, y: 50.88, z: 50.19 and box size x: 27, y: 27, z: 27.

### 3.4. Aromatase Enzymatic Activity Assay

Aromatase activity was tested using a fluorogenic assay from Abcam following the manufacturer’s instructions. The enzymatic activity was measured without (with DMSO) and with an increasing concentration (from 0 up to 100 μM) of any compound. Moreover, the aromatase enzymatic activity was also assessed in the presence of 1 μM of letrozole. The activity was measured for one hour at 37 °C, reading the emitted fluorescence every 50s for 1 h. The IC_50_ values were calculated using GraphPad Prism 9.0 (GraphPad Software).

### 3.5. Cell Culture

MCF 10A, MCF7 and T-47D cell lines were obtained from the American Type Culture Collection (ATCC, Manassas, VA, USA). MCF 10A was cultured in DMEM/F12 supplemented with 5% horse serum, 2 mM L-glutamine, 20 ng/mL epidermal growth factor, 10 µg/mL insulin, 0.5 mg/mL hydrocortisone, and 100 ng/mL. MCF7 was grown in DMEM supplemented with 10% fetal bovine serum (FBS) and 2 mM L-glutamine; T-47D cells were cultured in RPMI supplemented with 10% FBS and 2 mM L-glutamine. All the cell lines were maintained in a humidified atmosphere of 5% CO_2_ at 37 °C.

### 3.6. Anticancer Assay

#### 3.6.1. MTT Assay

MCF 10A cells were seeded at a density of 8000 (for the 24 h point), 7000 (for the 48 h point), and 6000 (for the 72 h point) cells/well in triplicate in a 96-well plate in 200 μL of growth media. MCF7 and T-47D were instead seeded at a density of 10,000 (for the 24 h point), 8000 (for the 48 h point), and 7.000 (for the 72 h point) cells/well in triplicate in a 96-well plate in 200 μL of growth media. After 24 h, the culture medium was replaced with media containing increasing concentrations (from 0 up to 100 μM) of **C4**, **C5**, **C6**, and **C7**. The plates were then incubated at 37 °C for 24 h, 48 h and 72 h. A measure of 20 μL of MTT solution (5 mg/mL) was added to each well and the cells were further cultured for another 2 h. The medium was then discarded, and the formazan crystals were dissolved with DMSO. The optical density was read at 470 nm using a Cytation 3 imaging reader (BioTek, Winooski, VT, USA). Cell proliferation rates were calculated by comparing the treated cells with the control cells (treated only with DMSO). Statistical significance was assessed using Student’s *t*-test. IC_50_ values were calculated using GraphPad Prism 9.0 (GraphPad Software, Inc., La Jolla, CA, USA).

#### 3.6.2. RNA Extraction and Real Time qPCR

MCF 10A, MCF7 and T-47D cells were plated in 6-well plates at a density of 280.000 cells/well. Twenty-four hours later, the cells were subjected to the treatment with 1 μM **C4**, **C5**, **C6** and **C7** for 24 h. The cells were lysed and the total RNA was extracted using RNeasy isolation kits (Qiagen, Hilden, Germany), and cDNA was obtained using QuantiTect Reverse Transcription kits (Qiagen, Hilden, Germany). The real-time qPCR was performed using the LightCycler^®^ 480 SYBR Green I Master (Roche, Indianapolis, IN, USA) and the LightCycler 480 Instrument II (Roche, Indianapolis, IN, USA). The real-time qPCR program consisted of an initial step of 15 min at 95 °C, and then 40 cycles, as follows: 95 °C for 10s, 60 °C for 30 s, and 72 °C for 30 s. glyceraldehyde-3-phosphate dehydrogenase (GAPDH) was used as a housekeeping gene. The mRNA levels in cells subjected to pharmacological treatments were quantified by measuring the fold change compared to the control levels, i.e., mRNA levels in cells treated with DMSO alone. Statistical significance was assessed using Student’s *t*-test.

#### 3.6.3. Wound Healing Assay

MCF 10A, MCF7 and T-47D cells were plated in 12-well plates at a density of 600,000 cells/well. Twenty-four hours later, the cells were completely confluent. Then, the confluent cultures were scratched using a 10 μL pipette tip and then incubated overnight in the presence of 1 μM **C4**, **C5**, **C6** and **C7** in media supplemented with 0.5% FBS. Images of the wounds were acquired at 0 h (immediately after the scratch) and after 24 h of incubation with compounds using an inverted Zeiss Axio Observer Z1 widefield microscope equipped with an AxioCam MRm grayscale CCD camera and controlled using ZEN pro software (Zeiss, Oberkochen, Germany, https://www.bioz.com/result/zen%20pro%20software/product/Carl%20Zeiss). The wound areas were quantified using ImageJ software (National Institutes of Health, Bethesda, MD, USA, https://imagej.net/ij/, 1997–2018) and the wound closure was calculated using the following equation:

$$\%WoundClosure=\left(\frac{A1-A2}{A1}\right)\times 100$$

In the equation, *A*1 is the area measured at time 0 and *A*2 is the area calculated after 24 h. Moreover, the percentage of wound closure in cells treated with compounds was quantified by measuring the fold change compared to the control wound closure, i.e., wound closure in cells treated with DMSO alone. Statistical significance was assessed using Student’s *t-*test.

#### 3.6.4. Gelatin Degradation (Invasion) Assay

Fluorescent-coated coverslips were prepared as described previously [38]. Cells were plated on gelatin-coated coverslips in a 24-well plate at a density of 70,000 cells/well. After 24 h, the cells were subjected to treatment with 1 μM **C 4**, **5**, **6** and **7** and fixed after a further 24 h with 4% paraformaldehyde (*v*/*v*) for 15 min at room temperature. Then, filamentous actin and nuclei were stained using Alexa Fluor™ 488 Phalloidin (Invitrogen, Carlsbad, CA, USA) or Hoechst 33,342 (Invitrogen, Carlsbad, CA, USA), respectively. Images were acquired using a confocal microscope (LSM 510; Zeiss, Oberkochen, Germany) and the degradation areas were quantified using ImageJ software. Statistical significance was assessed using Student’s *t*-test.

## 4. Conclusions

BC is still a global health problem, being one of the leading causes of cancer-related death worldwide. Among the different types of BC, hormone-dependent BC is the most common in post-menopausal women [1,2,3]. Despite research efforts and newly available therapies, there is still a need to develop more precise and efficacious therapy strategies to treat this malignancy. Aromatase inhibitors are now the first-line treatment for hormone-dependent BC in postmenopausal women, showing good results in the clinic [39]. However, aromatase inhibitors cause significant side effects including osteoporosis and heart issues [39]. For this reason, research is active in synthetizing and testing new molecules that inhibit the aromatase enzyme whilst having a safer profile with less severe side effects. In this paper, we report the synthesis and chemical characterization of four new AIs. The compounds are classified as non-steroidal Ais, and their chemical structures were determined using spectral analysis and microanalytical data. Through computational studies, we have demonstrated that the four compounds can bind aromatase, blocking its catalytic activity. This was confirmed by evaluating the ability of the four compounds to inhibit in vitro BC cell aromatase-dependent proliferation, migration and invasion activity. Moreover, the anti-cancer activity of the four compounds has been demonstrated to be specific as the compounds did not show any significant effects on healthy breast cells. Our work identifies four novel compounds with good profiles as anti-BC drugs. Future experiments will carried out to test these compounds in BC animal models.

## Figures and Tables

**Table 1:** Docking results of the synthesized compounds **1** (**C1**), **3** (**C3**), **4** (**C4**), **5** (**C5**), **6** (**C6**) and **7** (**C7**) and the co-crystalized ligand.

Compound	Binding Energy (Kcal/mol)	H Bonds	Residual Interactions
**C1**	−6.9	(2): Gly439-ala438	Leu152-ala307-met446-ala306 phe203-ile442
**C3**	−7.0	Gly439-ala438	Leu152-ala307-met446-ala306-phe203-ile442-ile132-cys437-val373
**C4**	−9.6	(2): Arg115-met374	Leu152-ala307-ala306-Leu372-Leu377-arg115-ile133-ile442-met311
**C5**	−10.1	(1): met374	Leu152-ala307-met446-ala306-phe203-ile442-arg115-Leu372-Leu377-ala343-val373-cys437-ala438-
**C6**	−9.7	(2): Arg115-met374	ala307-ala306-ala343-met364-cys437-Leu477-ala438-arg115-val373-ile442
**C7**	−9.8	(2): Arg115-met374	Leu152-ala307-met446-Leu372-Leu377-Leu372-Leu377-thr310-cys437-val370-met364-val370-ala443
(6alpha,8alpha)-6-(pent-2-yn-1-yloxy)androsta-1,4-diene-3,17-dione (Inhibitor)	−11.1	(2): Arg115-met374	Phe220-His480-val313-ile133-ala306

**Table 2:** IC_50_ values of **C4**, **C5**, **C6** and **C7** in MCF7 cells.

Compound	24 h	48 h	72 h
**C4**	3.722 μM	2.267 μM	1.072 μM
**C5**	3.255 μM	1.201 μM	1.228 μM
**C6**	3.086 μM	1.417 μM	1.501 μM
**C7**	2.692 μM	1.052 μM	1.22 μM

**Table 3:** IC_50_ values of **C4**, **C5**, **C6** and **C7** in T-47D cells.

Compound	24 h	48 h	72 h
**C4**	3.177 μM	1.685 μM	1.67μM
**C5**	3.119 μM	1.897 μM	1.5 μM
**C6**	3.066 μM	1.643 μM	1.1 μM
**C7**	1.854 μM	1.455 μM	1.317 μM

## Data Availability

Data is contained within the article and supplementary material.

## Supplementary Materials

The following supporting information can be downloaded at: https://www.mdpi.com/article/10.3390/ph17010088/s1, Chart S1: (a) ^1^H NMR spectrum of Compound **1**. (b) Magnification to the aromatic part of the ^1^H NMR spectrum of compound **1** showed the two doublet signals at 7.03 and 7.40 with coupling constant value *J* = 10.0 Hz; Chart S2: ^13^C NMR spectrum of Compound **1**; Chart S3: (a) ^1^H NMR spectrum of Compound **3**. (b) ^1^H NMR spectrum of Compound **3**. (c) Magnification to the aromatic part of the ^1^H NMR spectrum of compound **3** showed the two doublet signals at 7.09 and 7.48 with coupling constant value *J* = 10.0 Hz; Chart S4: ^13^C NMR spectrum of Compound **3**; Chart S5: (a) ^1^H NMR spectrum of Compound **4**. (b) Magnification to the part of the ^1^H NMR spectrum of compound **4** showed the three signals of the three methyl groups along with the singlet signal of the methoxy group; Chart S6: ^13^C NMR spectrum of Compound **4**; Chart S7: ^1^H NMR spectrum of Compound **5**; Chart S8: ^13^C NMR spectrum of Compound **5**; Chart S9: (a) ^1^H NMR spectrum of Compound **6**. (b) Magnification to part of the ^1^H NMR spectrum of Compound **6** showed triplet and quartet signals of the (COOCH_2_CH_3_) group protons along with the two methyl groups protons at 2.60 and 2.69 ppm in addition to the methoxy group protons at 3.89 ppm; Chart S10: ^13^C NMR spectrum of Compound **6**; Chart S11: ^1^H NMR spectrum of Compound **7**; Chart S12: ^13^C NMR spectrum of Compound **7**; Chart S13: FT-IR Spectrum of the Newly Synthesized Compounds **1**–**7**; Figure S1: Effects of compounds 4, 5, 6 and 7 on MCF7 and T-47D viability; Figure S2: Compounds **4**, **5**, **6** and **7** did not affect MCF 10A cell proliferation; Figure S3: Effects of compounds **4**, **5**, **6** and **7** on cell proliferation-related genes in MCF 10A cells; Figure S4: Compounds **4**, **5**, **6** and **7** do not inhibit breast cell migratory and degrading activity; Figure S5: Docking results of synthesized compounds **1** and **3** against the target human aromatase enzyme.
