# Supplementary material for: Towards a New Generation of Hormone Therapies: Design, Synthesis and Biological Evaluation of Novel 1,2,3-Triazoles as Estrogen-Positive Breast Cancer Therapeutics and Non-Steroidal Aromatase Inhibitors
Source: Pharmaceuticals (Basel). 2024 Jan 9;17(1):88. doi: 10.3390/ph17010088 (PMC10818821; doi:10.3390/ph17010088)
Supplement: Supplementary file 1 [file pharmaceuticals-17-00088-s001.zip › pharmaceuticals-2711904-supplementary.pdf]

Supporting Information

Towards a New Generation of Hormone Therapies: Design, Synthesis and Biological Evaluation of Novel 1,2,3-Triazoles as Estrogen-Positive Breast Cancer Therapeutics and Non-Steroidal Aromatase Inhibitors

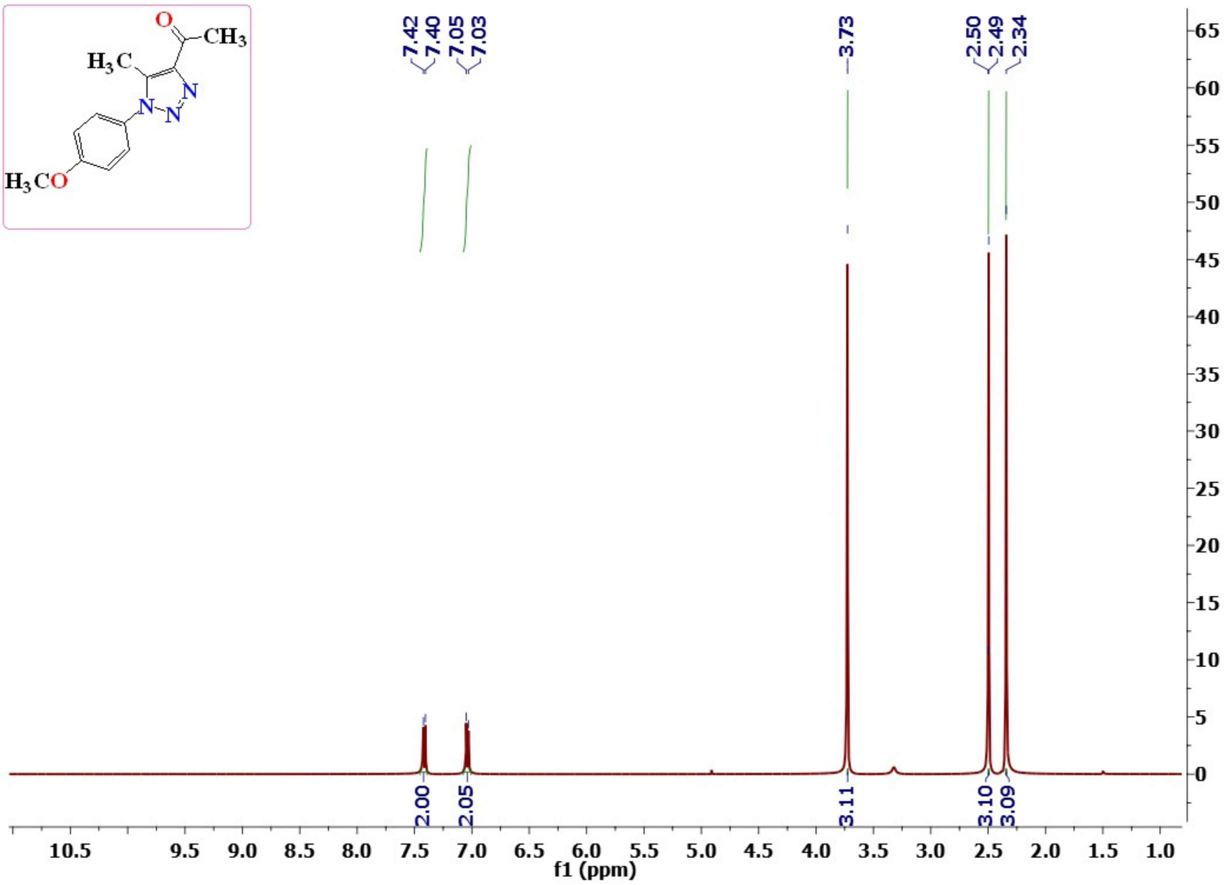

Chart S1a: H[1]-NMR spectrum of Compound **1**.

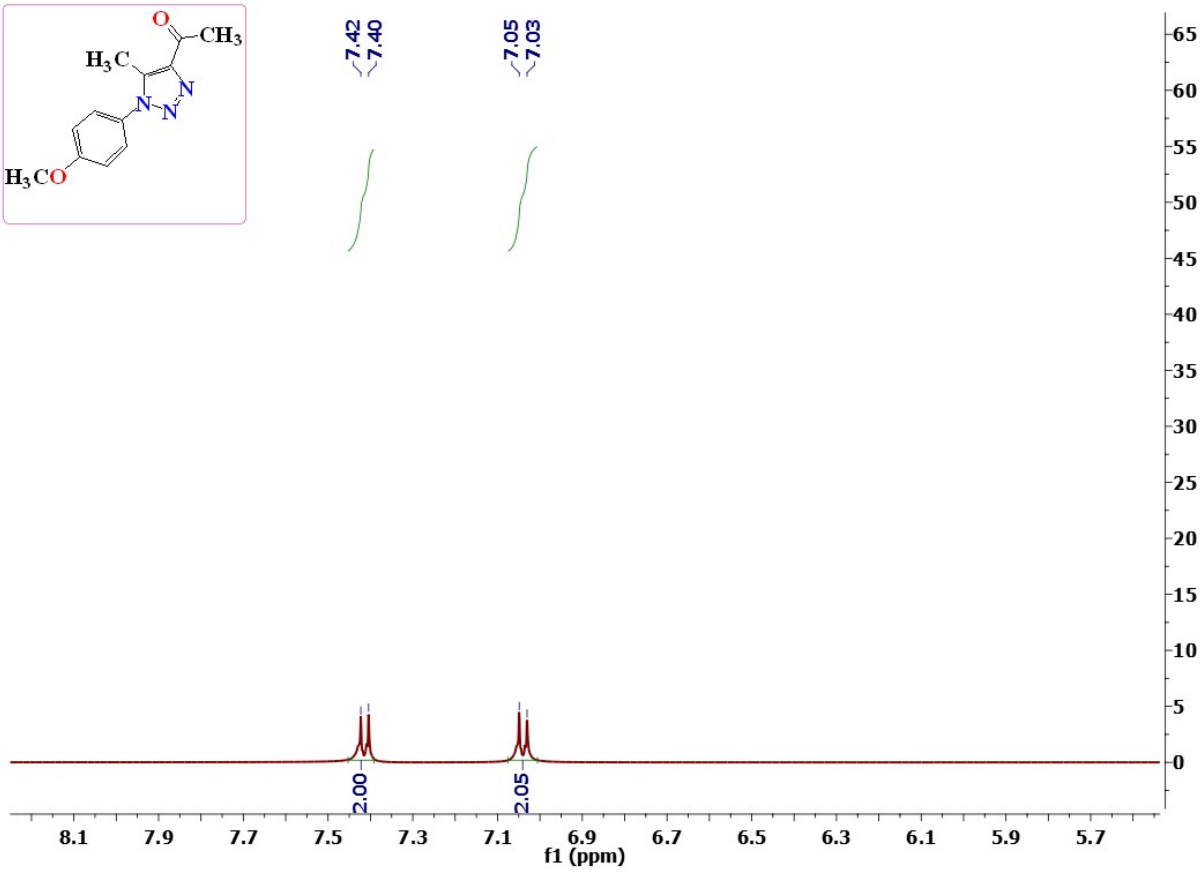

Chart S1b: Magnification to the aromatic part of the H[1]-NMR spectrum of compound **1** showed the two doublet signals at 7.03 and 7.40 with coupling constant value *J* = 10.0 Hz.

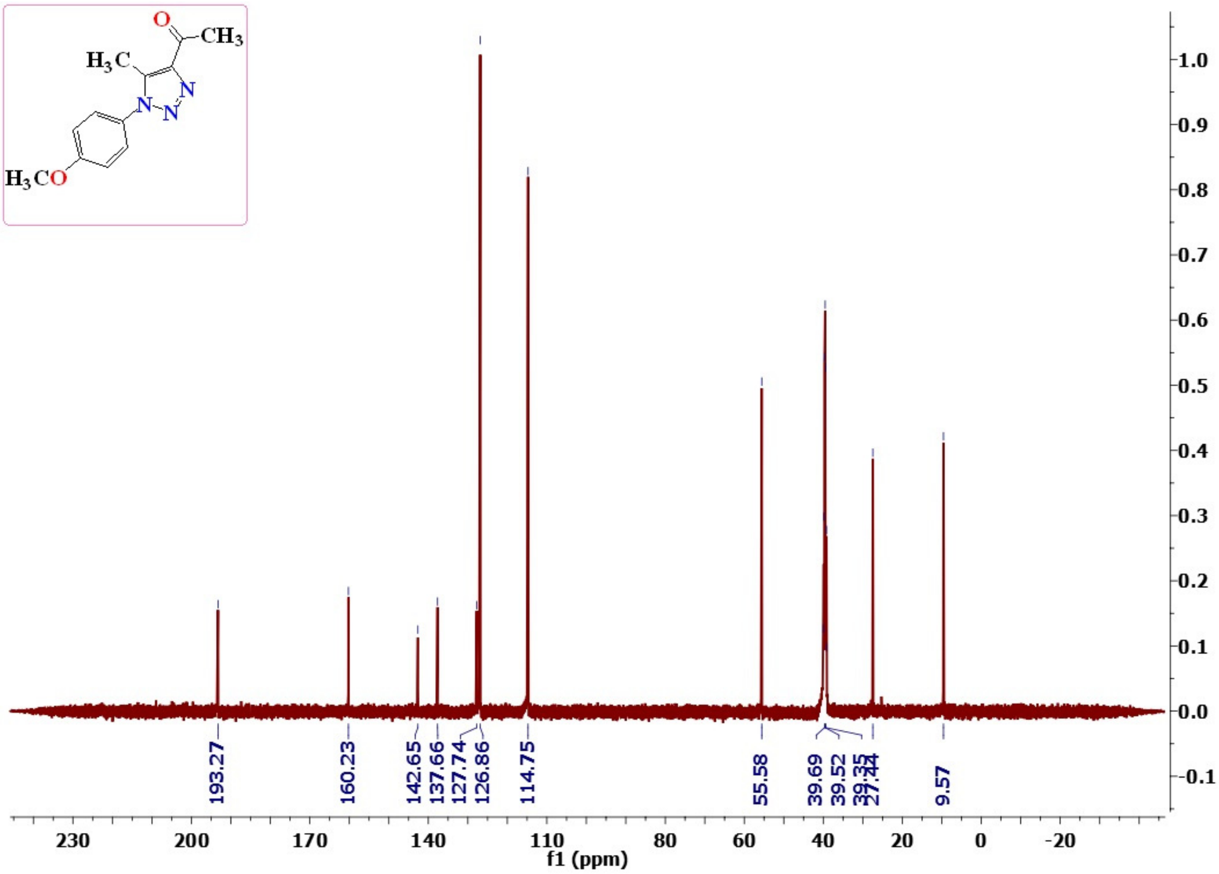

Chart S2: C^{13}-NMR spectrum of Compound **1**.

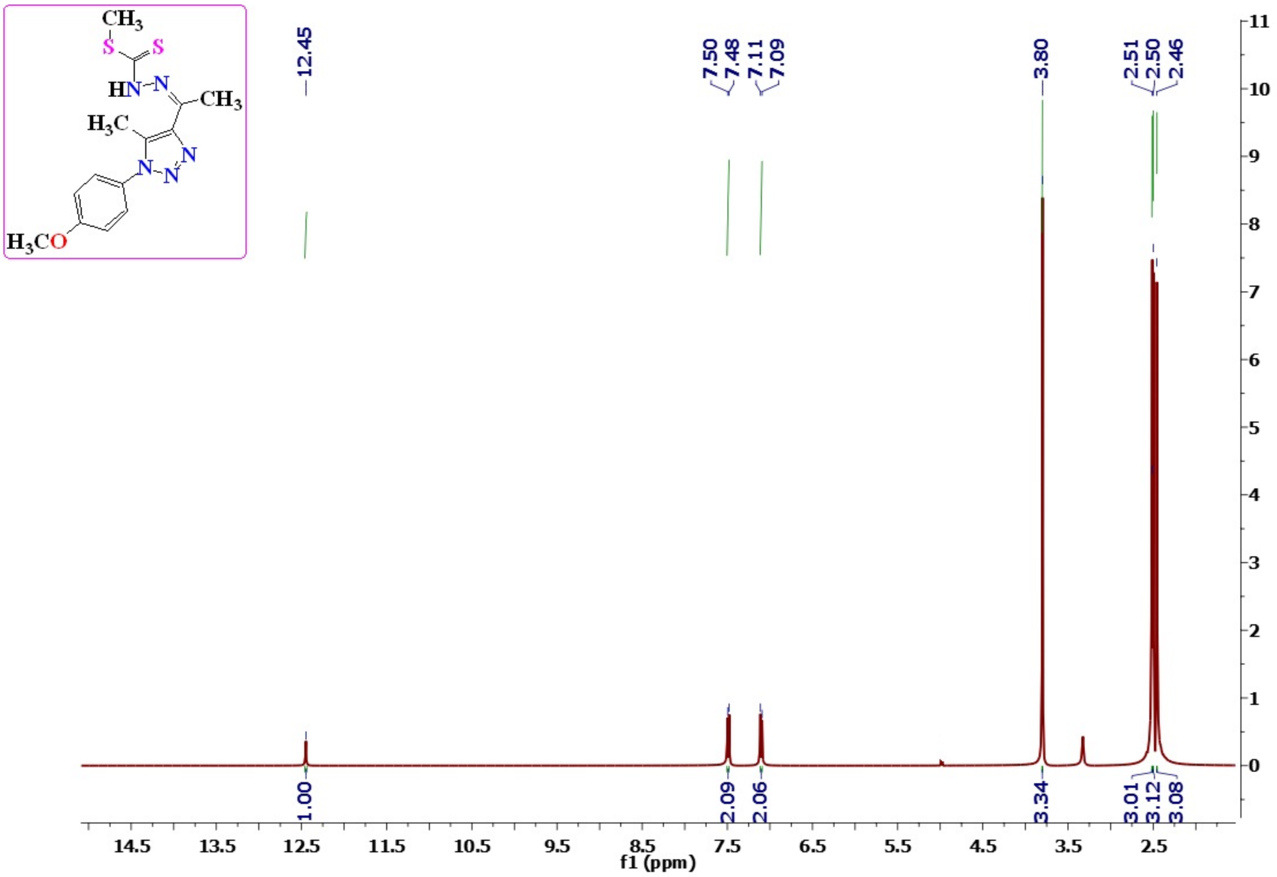

Chart S3a: H[1]-NMR spectrum of Compound **3**.

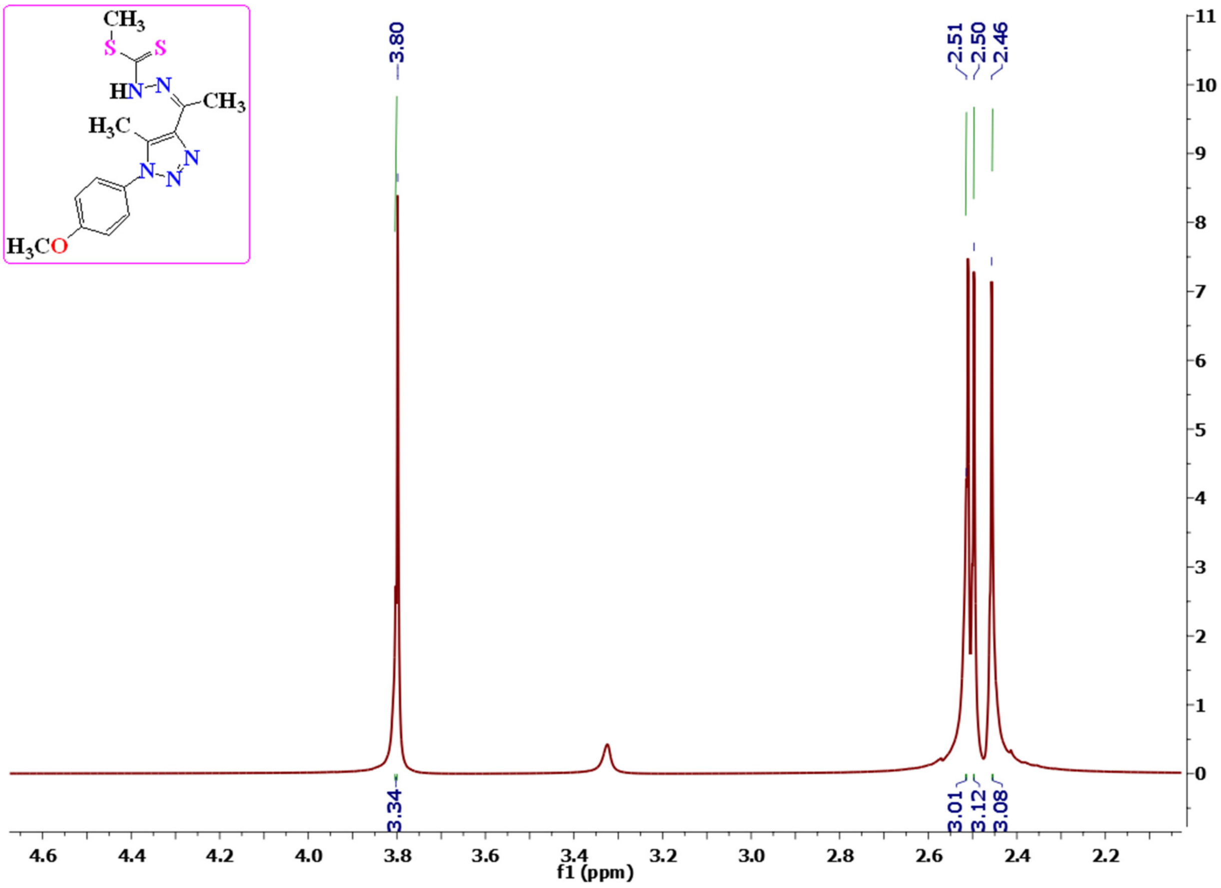

Chart S3b: H[1]-NMR spectrum of Compound **3**.

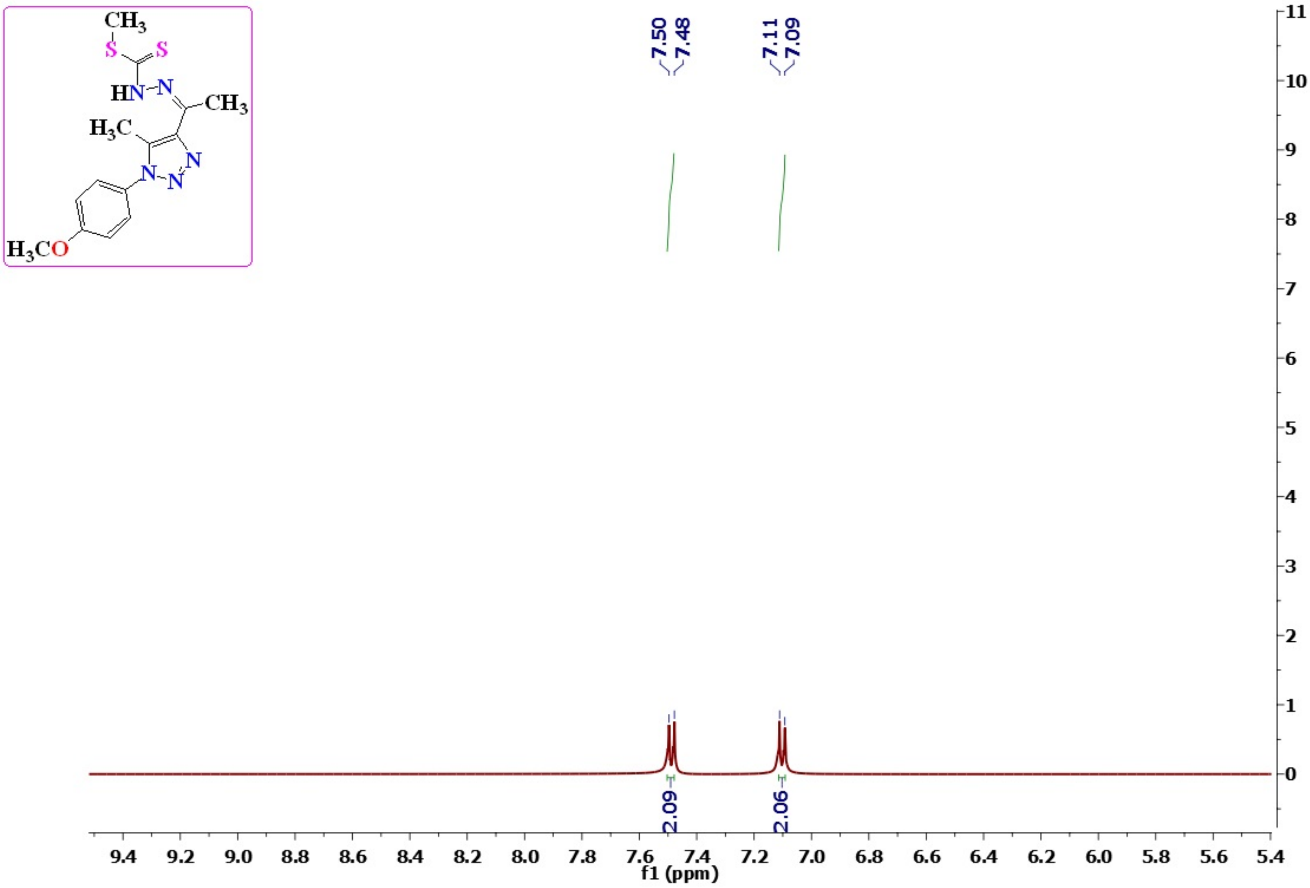

Chart S3c: Magnification to the aromatic part of the H^1-NMR spectrum of compound **3** showed the two doublet signals at 7.09 and 7.48 with coupling constant value *J* = 10.0 Hz.

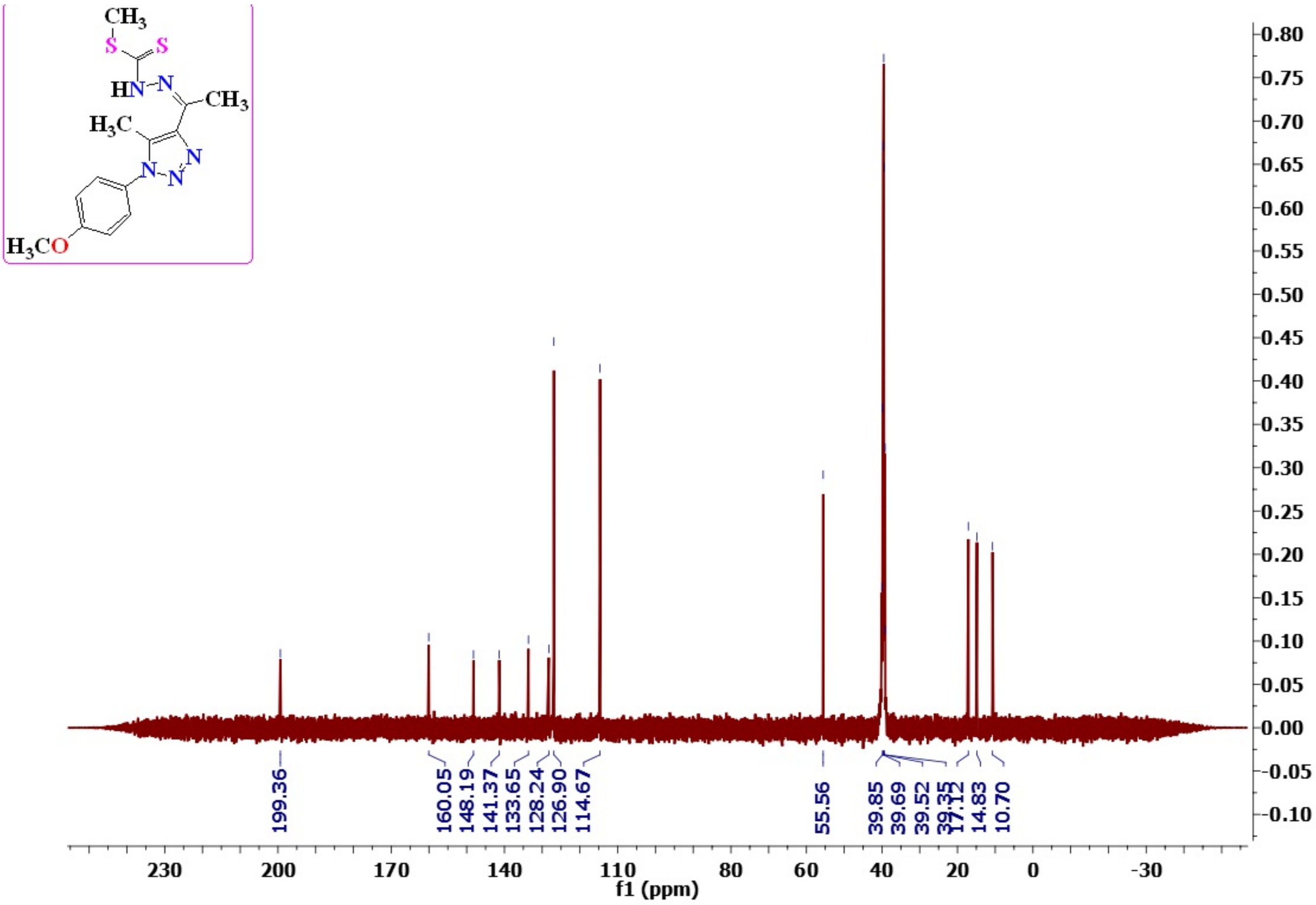

Chart S4: C13-NMR spectrum of Compound **3**.

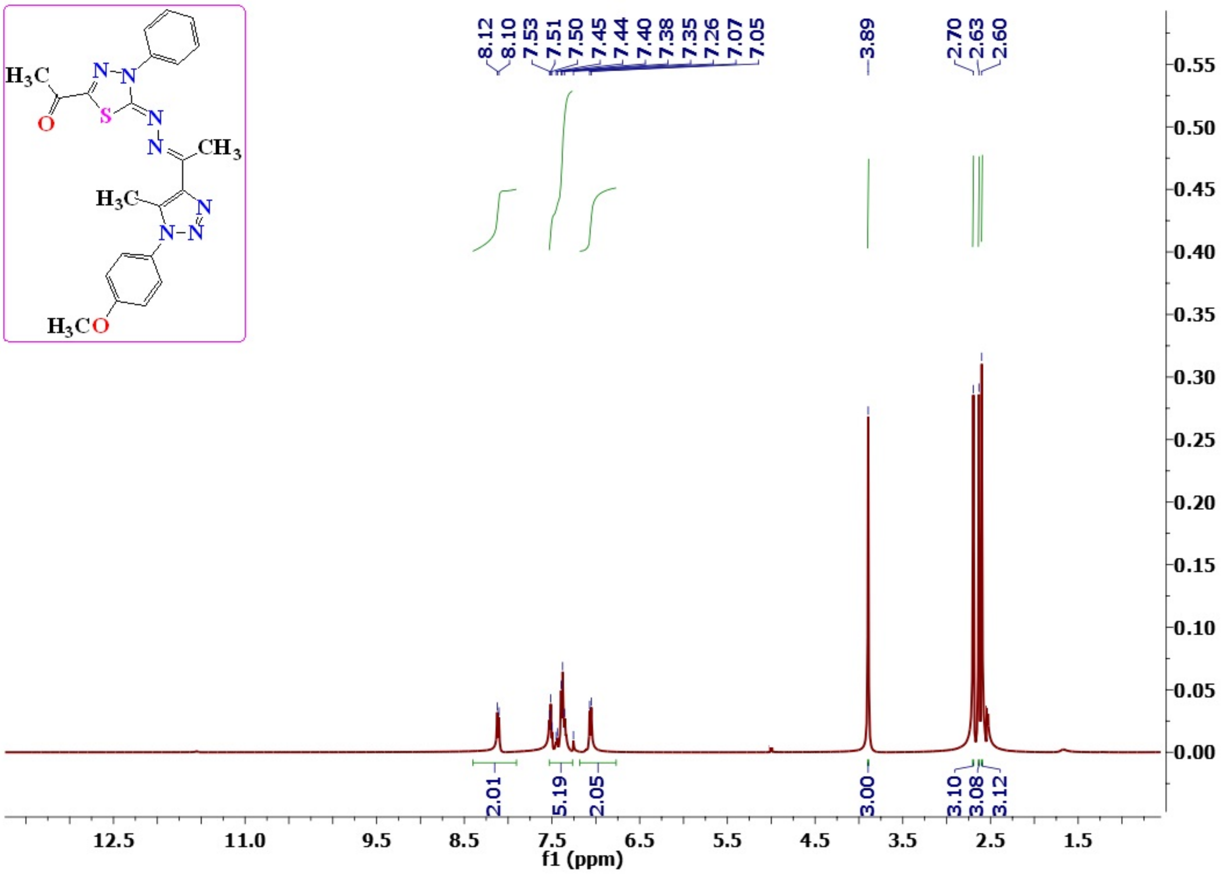

Chart S5a: H1-NMR spectrum of compound **4**.

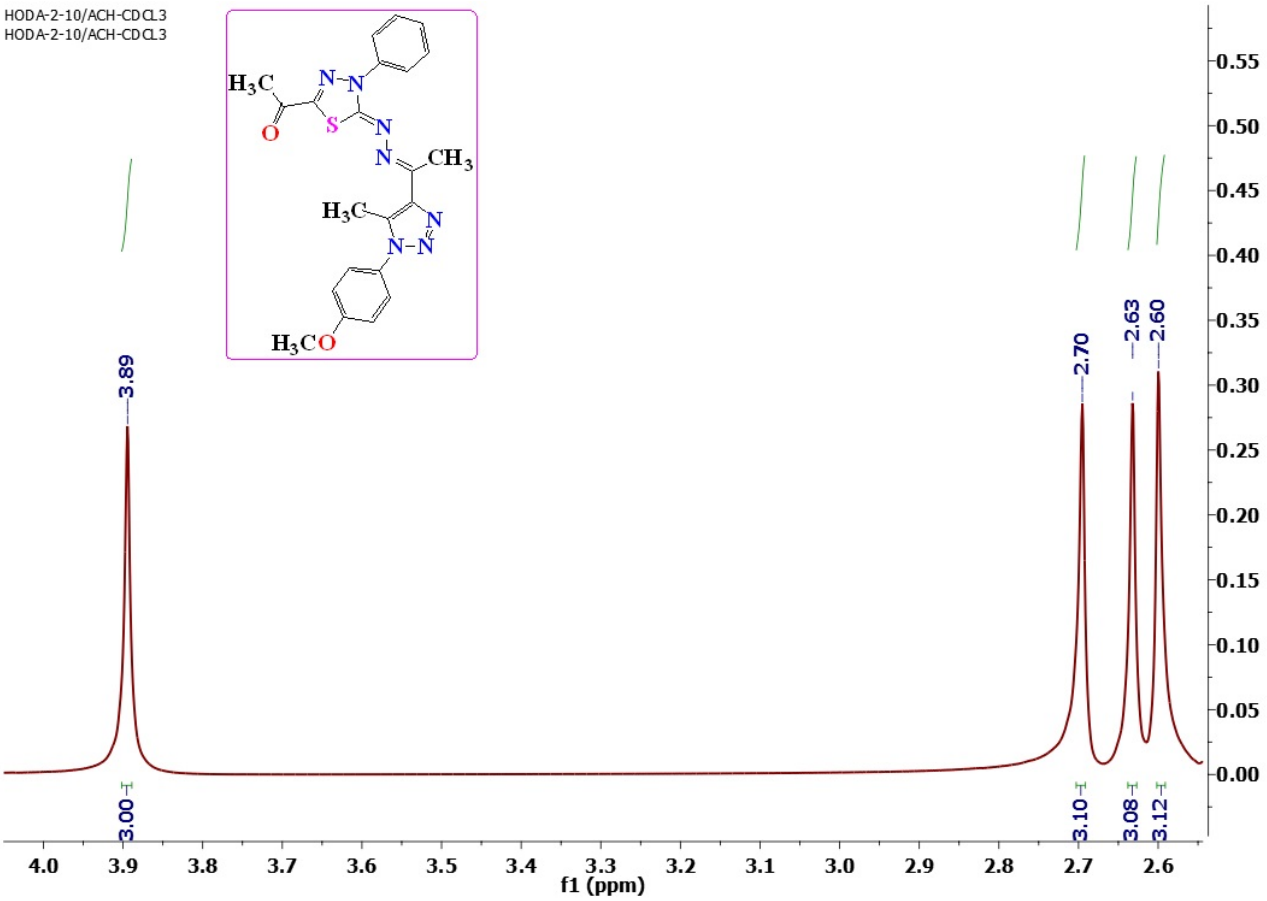

Chart S5b: Magnification to the part of the H[1]-NMR spectrum of compound **4** showed the thee signals of the three methyl groups along with the singlet signal of the methoxy group.

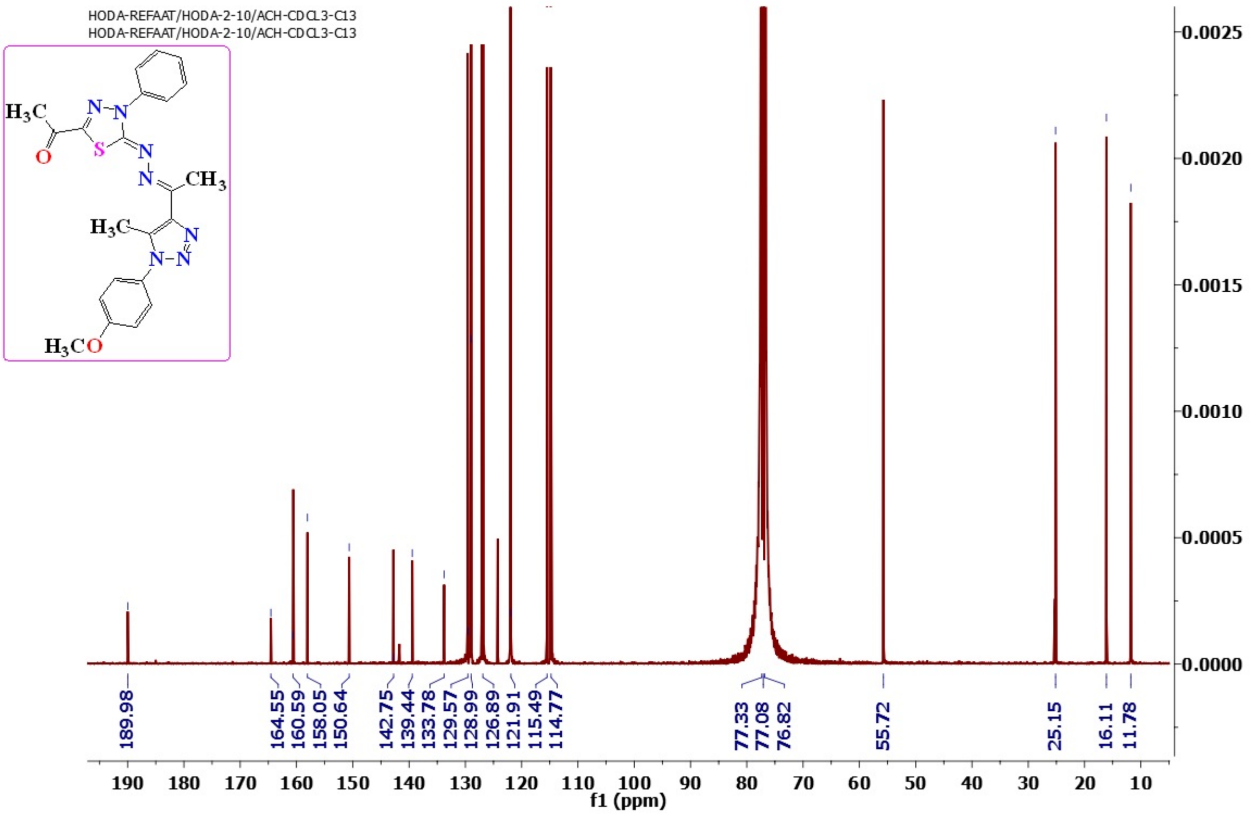

Chart S6: C13-NMR spectrum of Compound **4**.

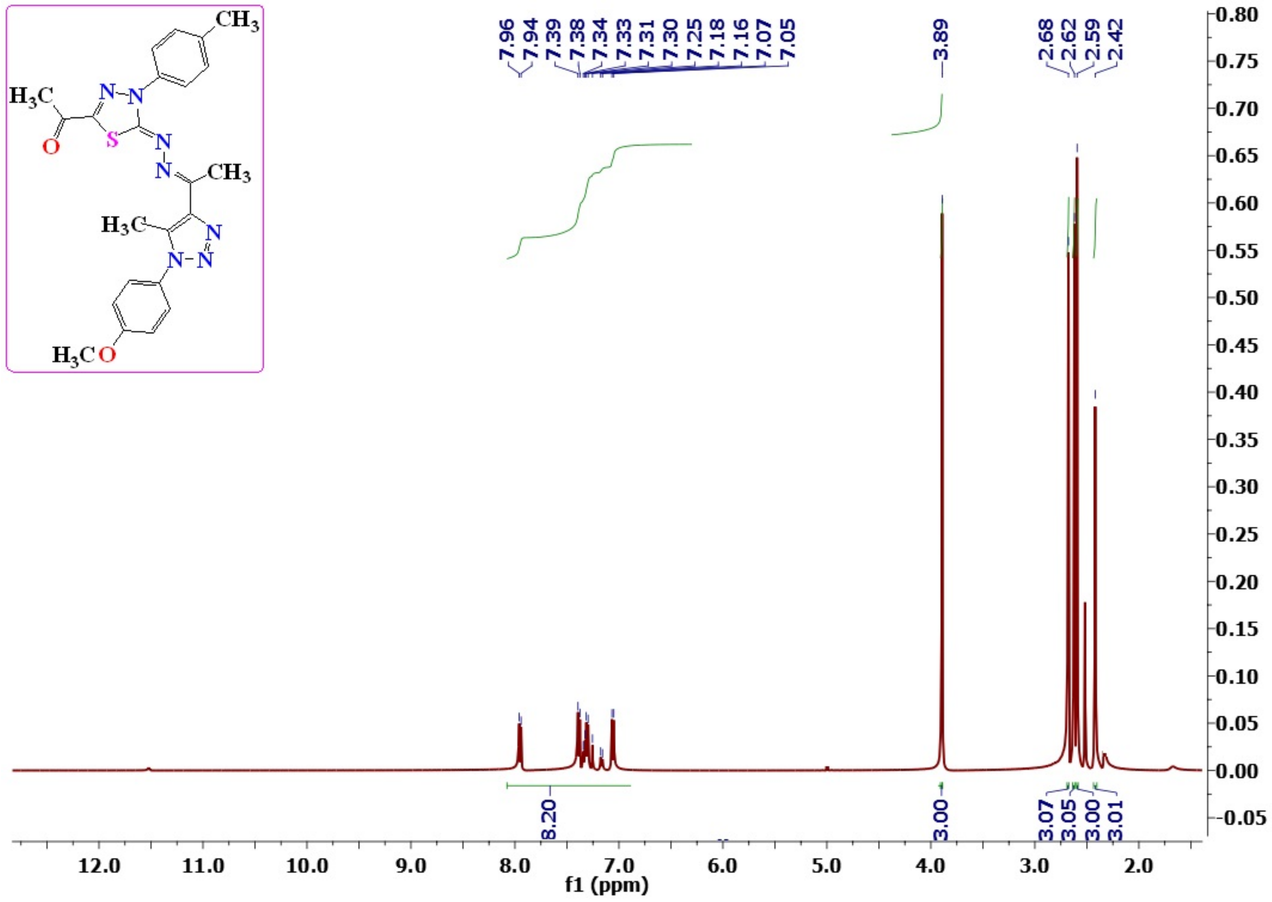

Chart S7: H[1]-NMR spectrum of compound **5**.

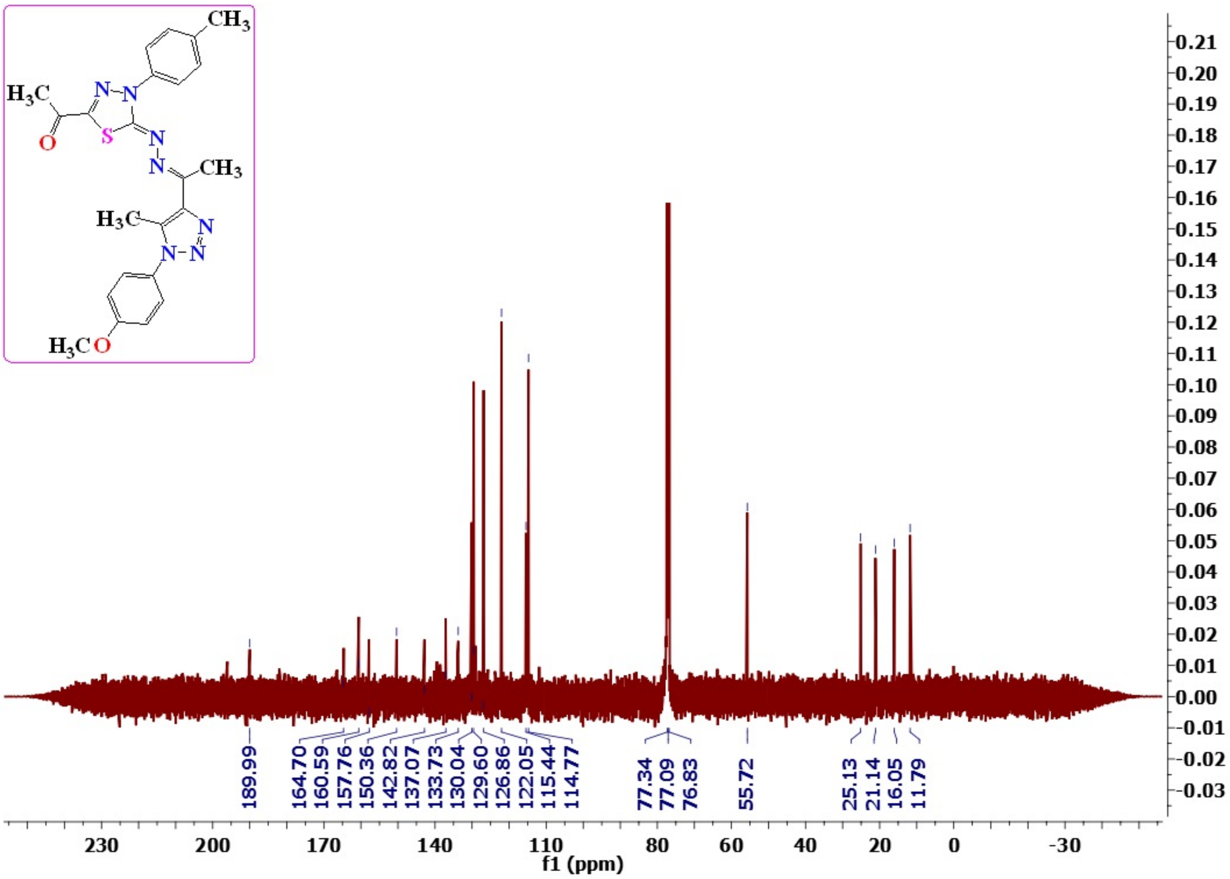

Chart S8: C^{13}-NMR spectrum of Compound **5**.

`

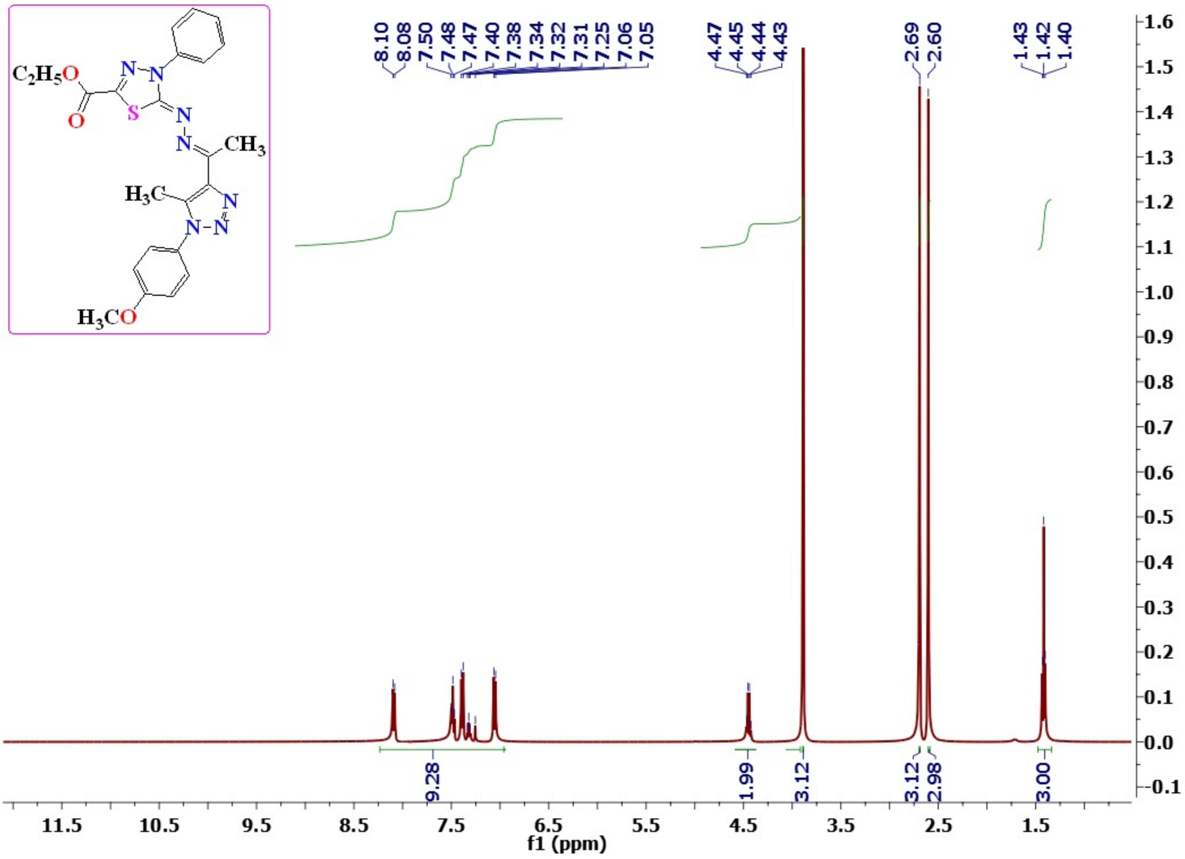

Chart S9a: H1-NMR spectrum of compound **6**.

.

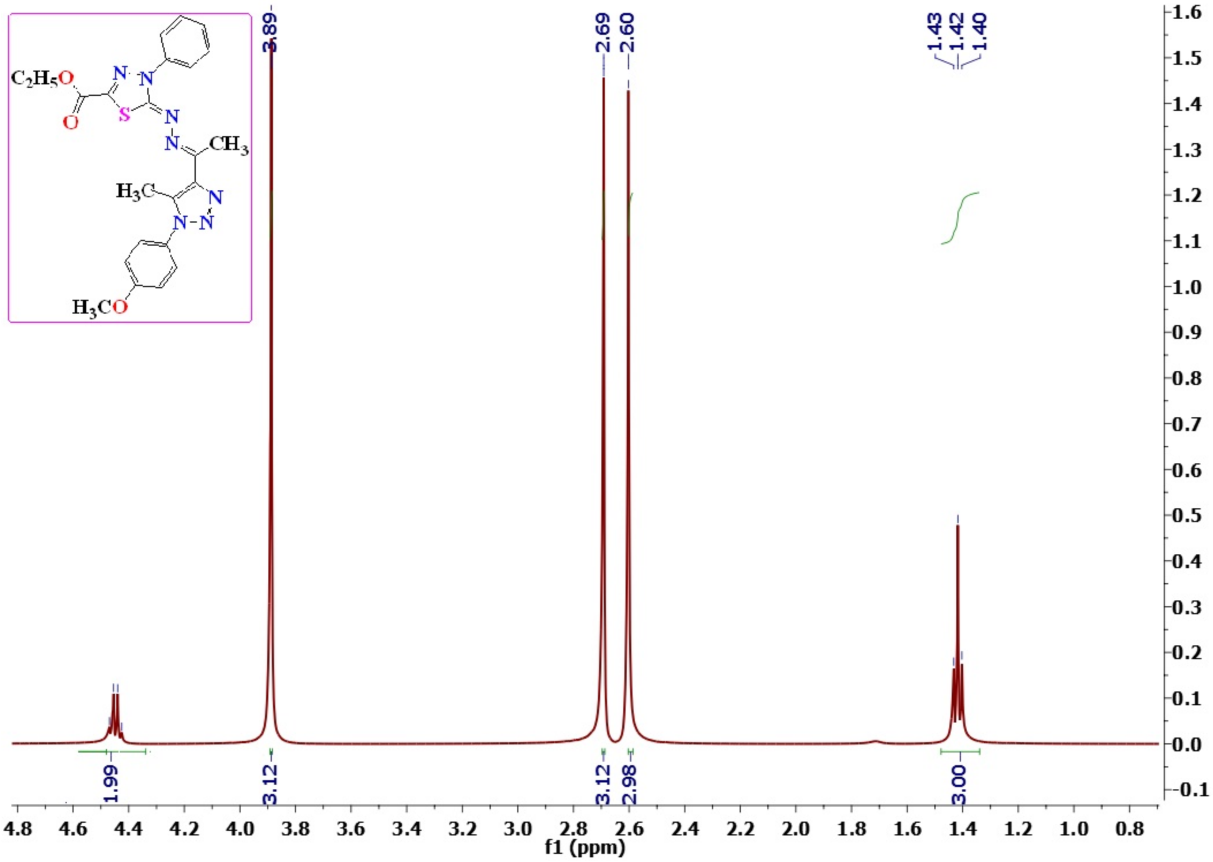

Chart S9b: Magnification to part of the H[1]-NMR spectrum of Compound **6** showed triplet and quartet signals of the ($COOCH_2CH_3$) group protons along with the two methyl groups protons at 2.60 and 2.69 ppm in addition to the methoxy group protons at 3.89 ppm.

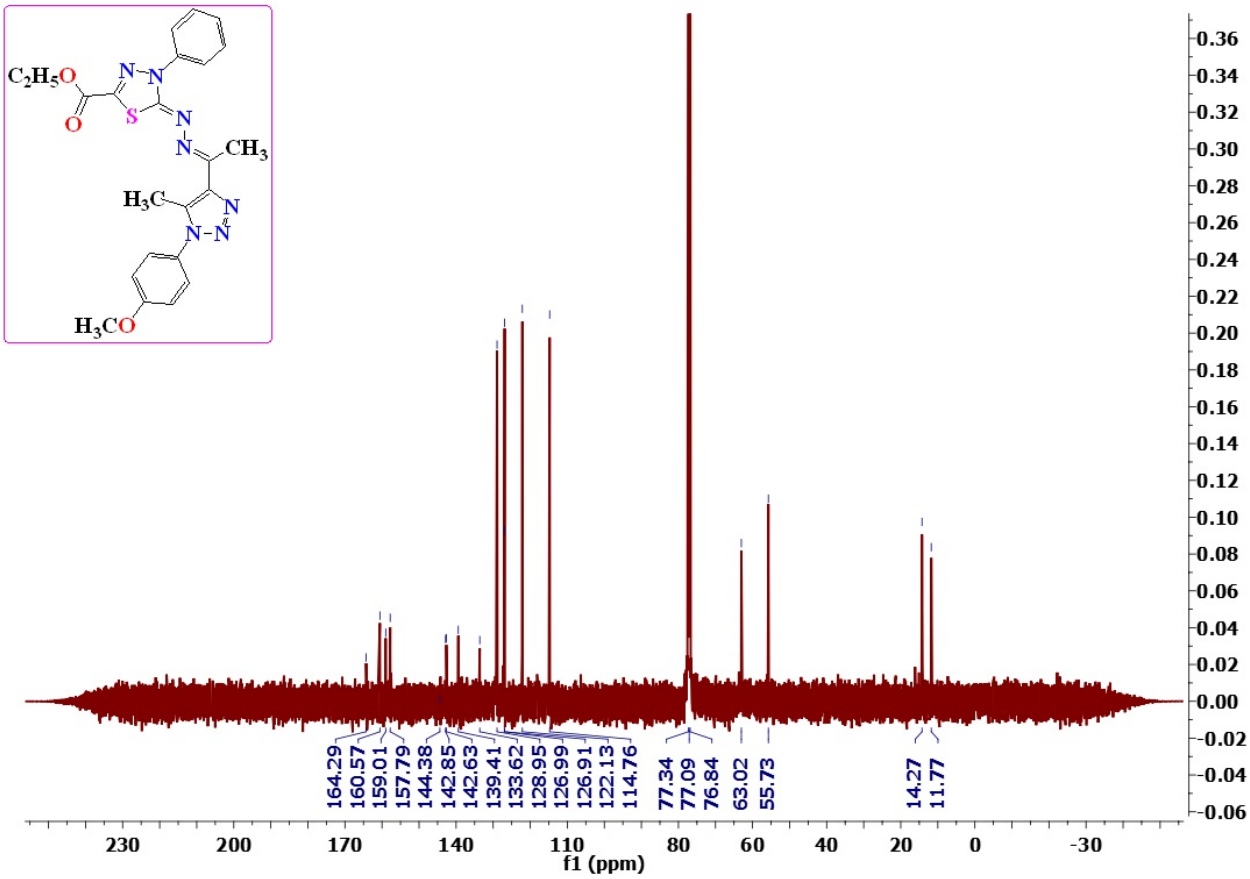

Chart S10: C^{13}-NMR spectrum of Compound **6**.

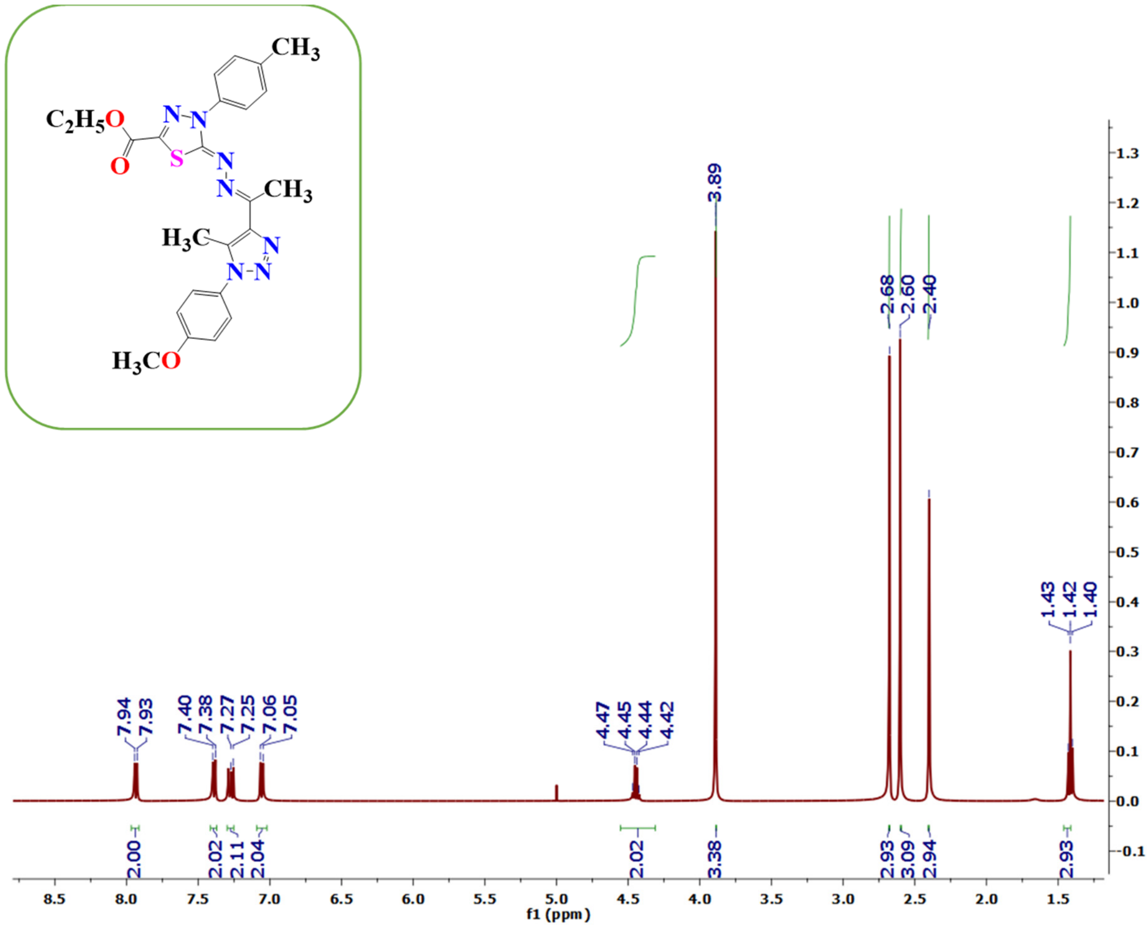

Chart S11: H[1]-NMR spectrum of compound **7**.

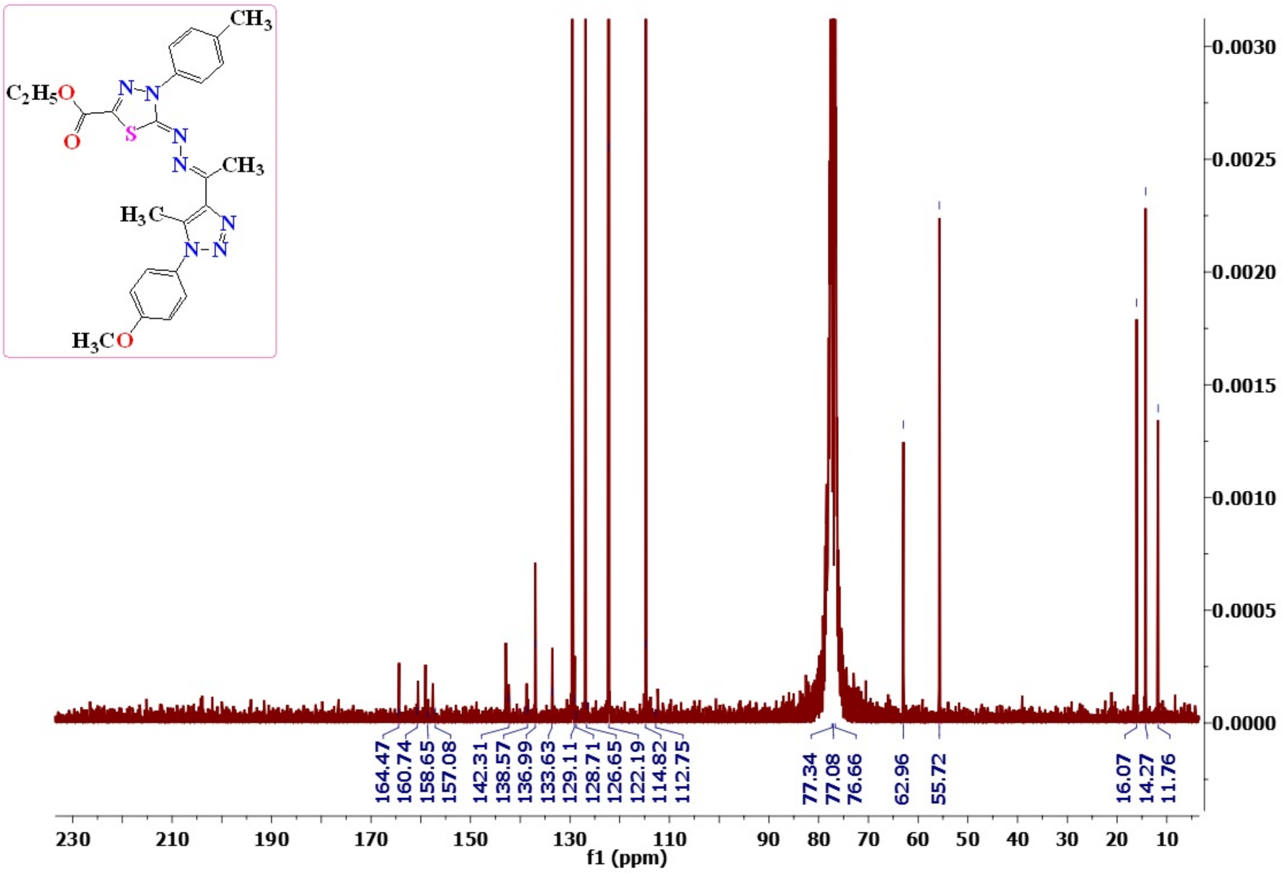

Chart S12: C^{13}-NMR spectrum of compound **7**.

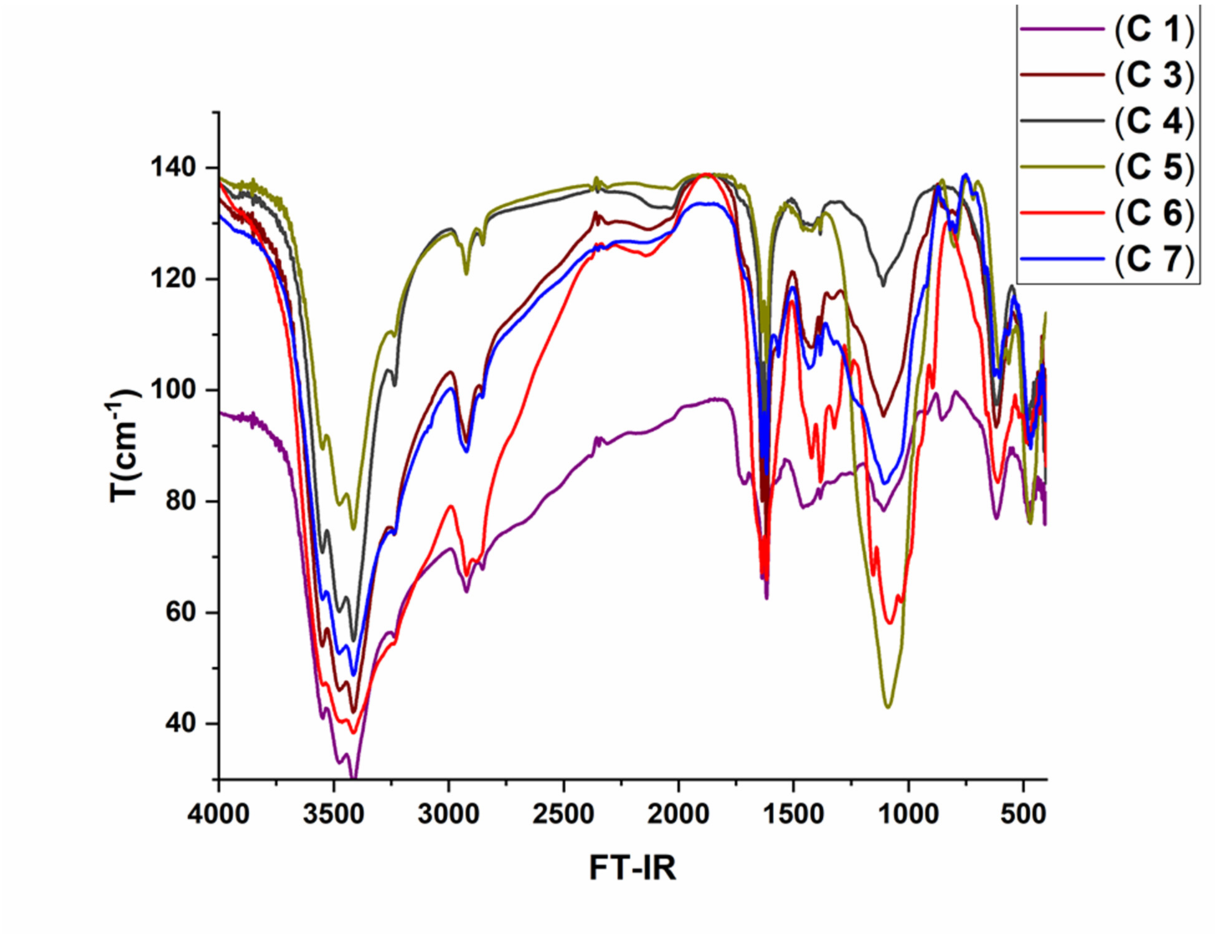

Chart S13: FT-IR Spectrum of the Newly Synthesized Compounds **1**–**7**.

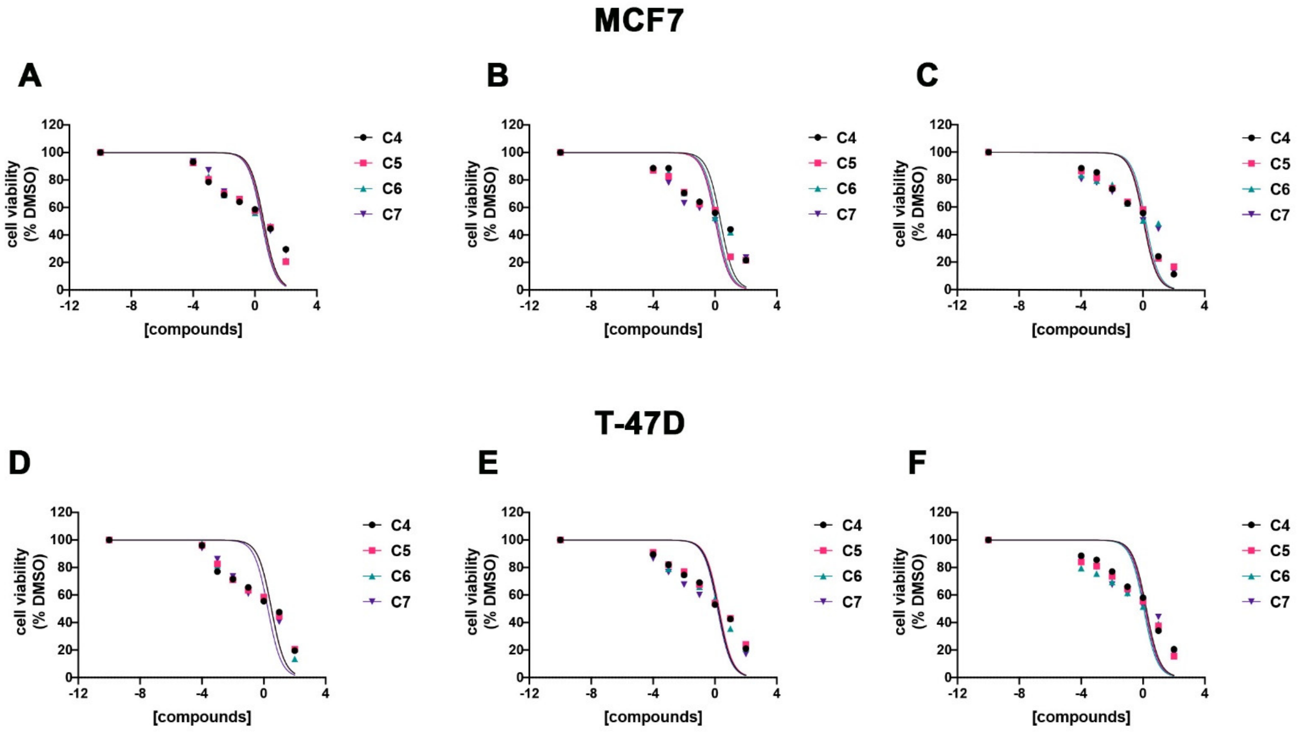

Figure S1. Compounds 4, 5, 6 and 7 effect on MCF7 and T-47D viability.
MCF7 (upper panels) and T-47D (lower panels) cells were grown in presence of increasing (from 0 to 100 μM) concentration of the compounds 4 (**C4**), 5 (**C5**), 6 (**C6**) and 7 (**C7**) for 24 (**A**, **D**), 48 (**B**, **E**) and 72 (**C**, **F**) hours. The cell viability was assessed performing the MTT assay. The graphs show the dose response of log10 concentration of the C4 (black dot), C5 (pink square), C6 (green triangle) and C7 (purple triangle) *versus* normalized optical intensity (570 nm). Data are shown as a mean ± SDs of three independent experiments.

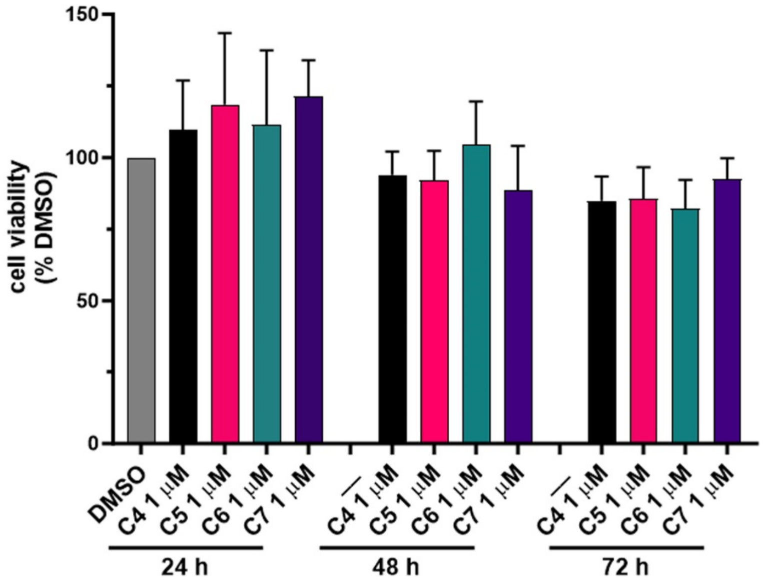

Figure S2. Compounds 4,5,6 and 7 did not affect MCF 10A cell proliferation.
Cell viability was assessed by MTT assay in normal cells (MCF10A) cells not treated (**DMSO**) cells or treated with 1μM of the compound **4** (**C4**), **5** (**C5**), **6** (**C6**, **panel C**), and **7** (**C7**) for 24, 48, and 72 hours (h). Data are showed as mean ± SDs of three independent experiments.

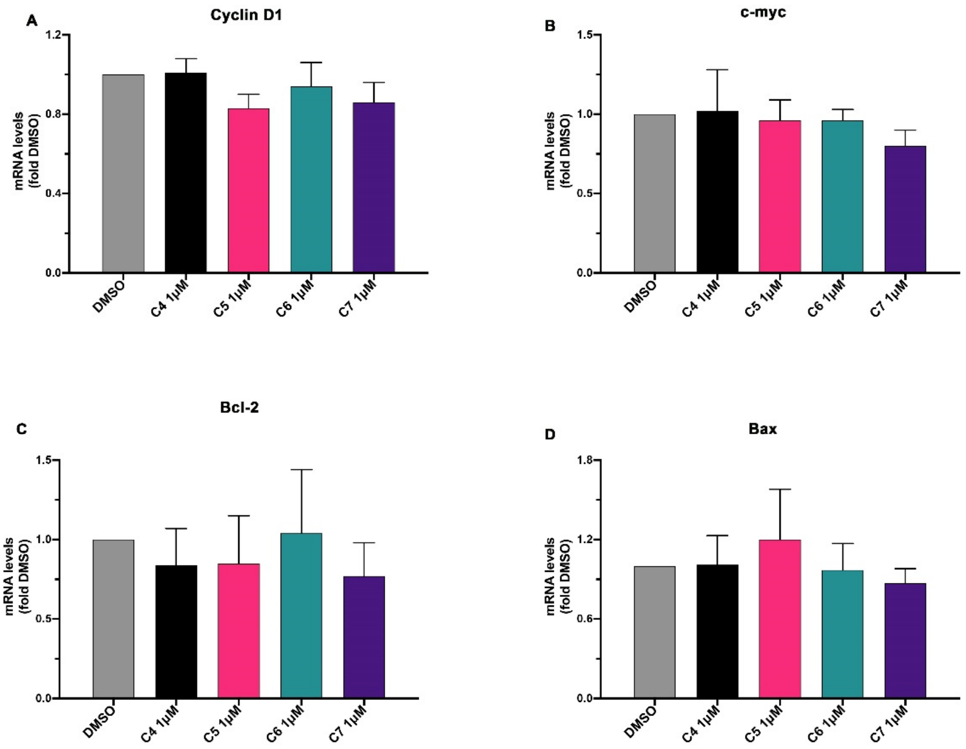

Figure S3. Compounds 4, 5, 6 and 7 effect on cell proliferation-related genes in MCF 10A cells.
MCF 10A cells were plated as described in Materials and Method and treated with DMSO or with compounds 4 (**C4**), 5 (**C5**), 6 (**C6**) and 7 (**C7**) at 1 µM for 24 hours. After the treatment, Cyclin D1 (**A**), c-myc (**B**), Bcl-2 (**C**) and Bax (**D**) gene expression were quantified by real-time qPCR and normalized using GAPDH expression as the housekeeping gene. Data are showed as mean ± SDs of three independent experiments.

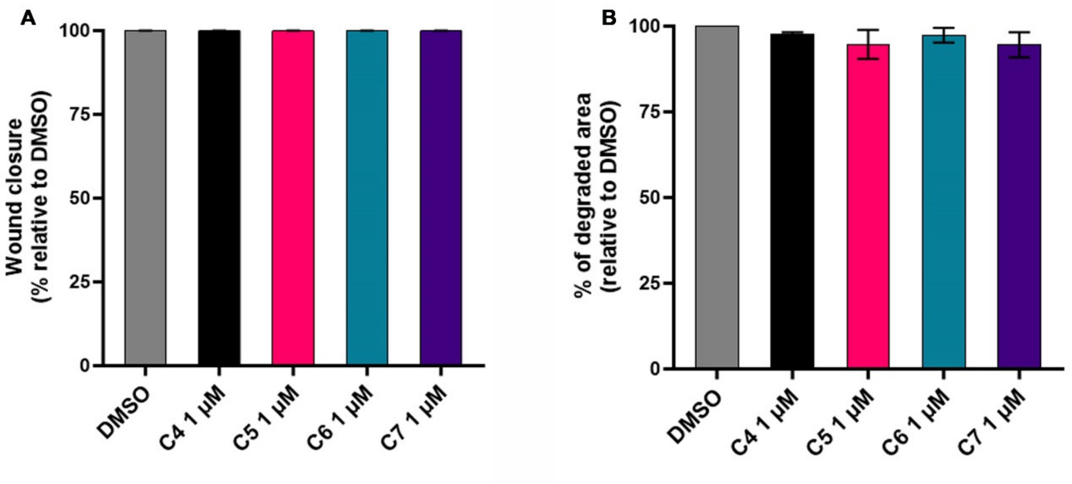

Figure S4. Compounds 4, 5, 6 and 7 do not inhibit breast cell migratory and degrading activity.
MCF 10A cells were plated as described in Materials and Method and treated with DMSO or with compounds 3 (**C3**), 4 (**C4**), 5 (**C5**) and 6 (**C6**) at 1 µM for 24 hours. The migratory capacity was assessed by wound healing assay (**A**) while the extracellular matrix degrading activity was evaluated through fluorescent gelatin degrading assay (**B**). After the treatment, the wound closure or the degraded area were calculated respect to the untreated cells (DMSO). Data are showed as mean ± SDs of three independent experiments.

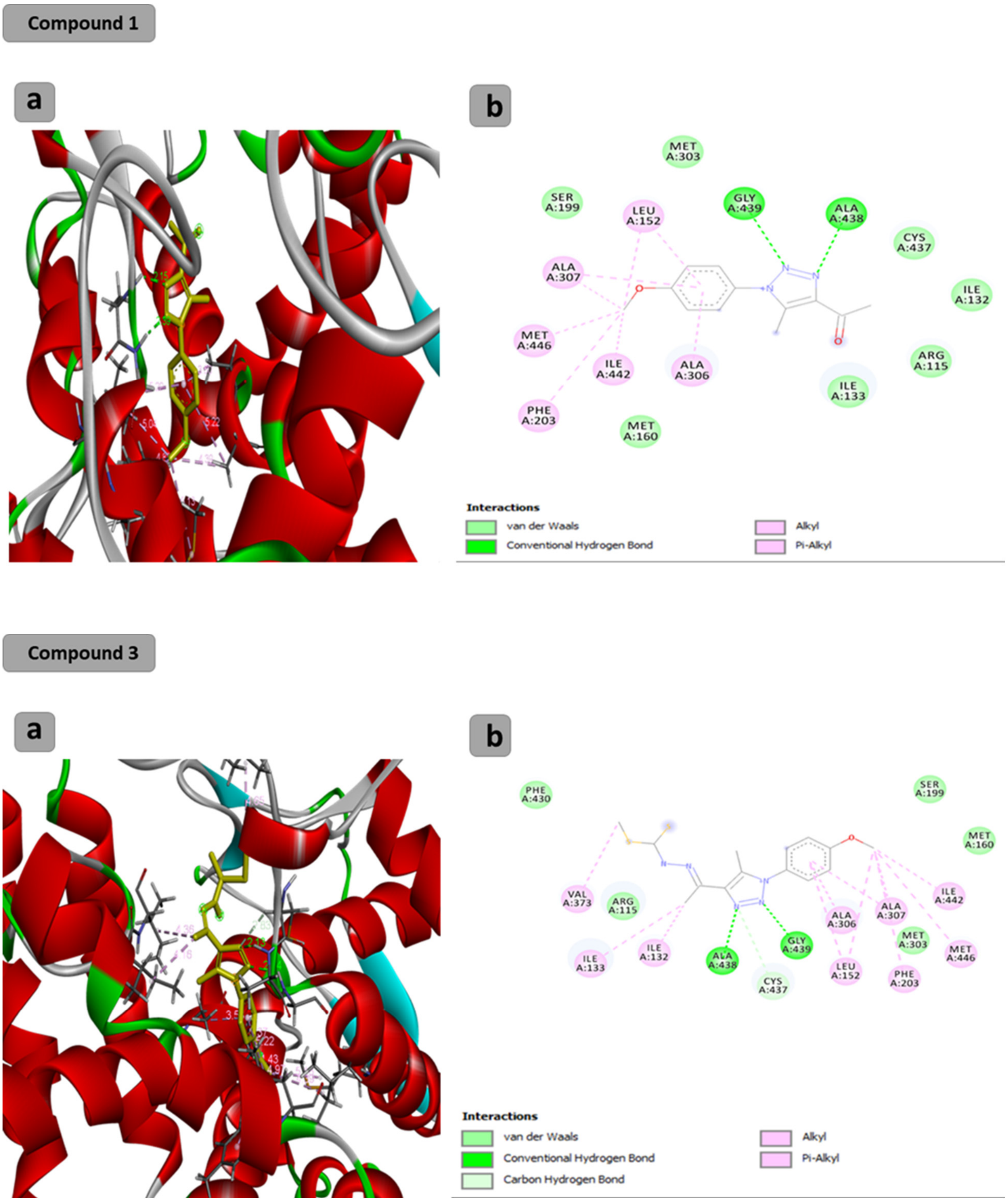

Figure S5. Docking results of the synthesized compounds **1 and 3**against the target human aromatase enzyme. (**a**) Three-dimensional (**3D**); (**b**) two-dimensional (**2D**) orientations of docked complexes. H-bond interactions are represented in blue and green dotted lines. Pi-stacked interactions are shown in orange lines.
